# *In vitro* models for gonadal and placental toxicity: A review and industry survey

**DOI:** 10.1016/j.namjnl.2025.100052

**Published:** 2025-09-13

**Authors:** Samuel Madureira Silva, Steven Van Cruchten, Freddy Van Goethem, Tamara Vanhaecke, Ellen Goossens, Yoni Baert

**Keywords:** *In vitro* toxicology, Gonadal toxicity, Placental toxicity, New approach methodologies (NAMs), Developmental and reproductive toxicology (DART), Steroidogenesis, Gametogenesis, Organ-on-a-chip, Microfluidic systems, Organoids, Animal-free testing, Regulatory toxicology, Survey study, Human-relevant models, Endocrine disruption

## Abstract

In recent years, there has been a significant push towards the development of animal-free methods in toxicology. Despite this progress, the adoption of such methods in safety assessment practices remains limited, particularly in the context of gonadal and placental toxicity. This paper reviews current *in vitro* models relevant to gonadal (gametogenesis and steroidogenesis) and placental biology, and potentially applicable in developmental and reproductive toxicology (DART). Additionally, we present the results of a survey conducted among DART experts (*n* = 16), examining current practices and perceptions in industry regarding these *in vitro* methodologies. The findings indicate a predominant reliance on animal models, largely driven by regulatory requirements, despite concerns about their ability to reliably predict human outcomes. Respondents reported limited familiarity with and confidence in available *in vitro* models for gonadal and placental toxicity, yet expressed optimism about their future integration. These findings underscore the need for increased awareness of gonadal and placental *in vitro* models, particularly among DART risk assessors, to facilitate a shift toward more reliable and human relevant risk assessments.

## Highlights

• Review of *in vitro models* for gonadal and placental toxicity in DART.
• Survey of 16 industry experts reveals limited use and confidence in current *in vitro* models.
• Regulatory demands remain a key barrier to NAM adoption in reproductive toxicology.
• Industry shows optimism for future integration of *in vitro* models.
• Recommendations provided to support broader acceptance of NAMs in DART.

## Introduction

The continuous growth in the production and use of chemicals, pharmaceuticals, and cosmetics has intensified the demand for robust toxicity assessment strategies to safeguard human health and the environment. Toxicity assessment is essential for identifying potential adverse effects of compounds, informing regulatory decision-making, and ensuring product safety.

In recent years, there has been a global push towards the development and implementation of New Approach Methodologies (NAMs), including *in vitro* models, to reduce reliance on animals for toxicity testing. Regulatory agencies have begun to outline and publish roadmaps for the integration of NAMs into safety assessment frameworks (Cronin et al., 2025; U.S. Food and Drug Administration, 2025; European Commission, 2025; European Commission, 2025). However, the adoption of *in vitro* methodologies within the field of developmental and reproductive toxicology (DART) remains limited. In particular, gonadal and placental toxicities, critical for understanding reproductive function, endocrine disruption, and maternal-foetal interactions, are notably underrepresented in this context (International Council for Harmonisation, 2020; Niethammer et al., 2022).

This review explores the current landscape of *in vitro* models for assessing gonadal and placental toxicity, with an emphasis on perspectives from industry stakeholders. Drawing on a survey conducted among experts in the DART field, we examined the level of familiarity with and confidence in these models across different sectors, including the pharmaceutical, agrochemical, cosmetic industries, as well as contract research and/or method development organizations. By outlining both the existing challenges and emerging opportunities, this review aims to support ongoing efforts to promote the adoption of more human-relevant, ethical, and scientifically robust testing strategies.

## Toxicity assessment of chemicals, pharmaceuticals, and cosmetics

The global production and sale of chemicals have increased consistently over the last decades (European Chemical Industry Council (Cefic), 2018; Landrigan et al., 2020; Naidu et al., 2021)). In this context of rising and sustained exposure to both new and persistent chemical agents, toxicity assessment has become a critical scientific discipline, aimed at evaluating the potential adverse effects of substances on living organisms and the environment.

Although pharmaceuticals may share structural similarities with industrial chemicals, they are typically considered as a separate category. They are deemed more tolerable regarding adverse effects as long as these do not outweigh the therapeutic benefits. Yet, in the search for new and effective pharmaceuticals, toxicity assessment is a critical step by identifying unsuitable candidates early in the development pipeline and safeguard the health of prospective patients (Pognan et al., 2023; Beilmann et al., 2025). This fundamental difference divides chemicals and pharmaceuticals into distinct regulatory frameworks across various global jurisdictions (International Council for Harmonisation, 2025; European Chemicals Agency, 2006; U.S. Environmental Protection Agency, 2025). Additionally, cosmetics may follow their own regulatory framework (European Parliament and Council, 2009; Government of Canada, 1978) or be subject to specific provisions within broader frameworks (United States Congress, 1938). These can overlap with chemical regulations, especially when considering new ingredients and production volume thresholds, or with pharmaceutical regulations when therapeutic or medical claims are intended. Another important aspect regarding cosmetics is that, like in the EU, most regulatory frameworks ban the use of animals for their safety assessment (Pistollato et al., 2021). As a result, the cosmetics industry has emerged as a forerunner in the development and application of non-animal testing methods.

## New approach methodologies

Mechanistic, or investigative, toxicology is pivotal in understanding the molecular mechanisms through which substances cause adverse health effects. *In vitro* models have increasingly demonstrated to be more suitable than animals for this purpose and are in growing demand (Pognan et al., 2023; Beilmann et al., 2025; Bernauer et al., 2005; Lynch et al., 2019; Anadón et al., 2014; Stoykova, 2025; Marshall et al., 2023). Notably, between 2019 and 2023, the European Medicines Agency’s (EMA) Innovation Task Force saw a steady increase in briefing meeting requests related to the 3Rs (Replacement, Reduction, and Refinement of animal use) in medicine development, with approximately 80 % of these requests focused on replacing specific animal tests with New Approach Methodologies (NAMs) (Edwards et al., 2025). EMA published a guideline describing the submission and evaluation process for NAMs proposed for regulatory use in the development of medicinal products, including the scientific and technical criteria required for validation (European Medicines Agency, 2016). Despite this progress, risk assessors across various sectors – including regulatory authorities, scientific organisations, consultancy firms, industry and academia – report significant limitations in their ability to choose how to conduct risk assessment due to existing legal requirements. This constraint persists even as there is broad consensus that NAMs should play a more prominent role in regulatory toxicology (Bearth et al., 2025).

At the same time, regulatory agencies worldwide are facing growing societal pressure to move away from animal testing, driven by environmental, ethical, and human health concerns (European Parliament and Council, 2021; Hansell et al., 2024; European Commission, 2023; U.S. Food and Drug Administration, 2021; European Citizens’ Initiative, 2021; European Citizens’ Initiative, 2012; Physicians Committee for Responsible Medicine, 2024; Niemiec et al., 2024; Savanta ComRes, 2023). This pressure is increasingly reflected in public campaigns and policy initiatives, such as the European Union’s ban on animal testing for cosmetics (Pistollato et al., 2021) and the “Save Cruelty-Free Cosmetics” European Citizens’ Initiative (European Citizens’ Initiative, 2021), which gathered widespread support across member states. Additionally, surveys consistently show that the public favours non-animal methods (Physicians Committee for Responsible Medicine, 2024; Niemiec et al., 2024; Savanta ComRes, 2023). These movements underscore a growing societal consensus that animal-free testing methods are not only preferable but necessary. Ethical considerations, including the principles of the 3Rs (European Medicines Agency, 2016), are also gaining traction in regulatory discourse and industry practice. Together, these factors are accelerating the demand for validated *in vitro* models that can meet both scientific and societal expectations.

Reflecting this shift, the United States Food and Drug Administration (FDA) recently released a “Roadmap to Reducing Animal Testing in Preclinical Safety Studies”, which envisions a future where animal testing becomes optional rather than mandatory (U.S. Food and Drug Administration, 2025). In parallel, next to EMA’s guideline on the regulatory acceptance of NAMs, the European Commission is advancing its own roadmap “Towards Phasing Out Animal Testing for Chemical Safety Assessments” covering the chemical, pharmaceutical and food safety sectors (Cronin et al., 2025; European Commission, 2025; European Commission, 2025).

NAMs encompass a range of tools, including computational models (*in silico*), chemical assays (*in chemico*), and cellular or tissue cultures (*in vitro*), all aimed at replacing, wholly or partially, traditional *in vivo* animal methods. Additionally, omics technologies can contribute to the refinement of animal use by providing deeper mechanistic insights into biological responses. Among these approaches, *in vitro* cultures are particularly valuable for their capacity to generate novel, experimentally-derived biological data, making them central to the advancement of human-relevant toxicological assessment (Beilmann et al., 2025).

Recent advances in artificial intelligence (AI) and genome editing technologies are opening new avenues in the development of NAMs. AI-integrated *in silico* models, including machine learning algorithms and deep neural networks, are being applied to predict toxicological outcomes based on chemical structure, omics data, and biological pathway modelling (Lin and Chou, 2022; Kleinstreuer and Hartung, 2024). These approaches can enhance the predictive power of NAMs by identifying complex patterns and interactions that may not be evident through traditional methods. CRISPR/Cas9 gene editing is also being used to engineer organoids and cell lines with specific genetic traits (Geurts and Clevers, 2023; Mukhare et al., 2025), enabling to test experimentally molecular initiating events and key events within adverse outcome pathways (AOPs), helping to establish causal links between chemical exposures and observed toxic effects. For example, targeted gene knockouts can be used to confirm whether a specific receptor or pathway is essential for mediating toxicity, thereby strengthening the mechanistic basis of NAMs.

## Assessment of developmental and reproductive toxicities

Development and reproduction are particularly susceptible to toxic insult due to factors such as rapid cell division and differentiation, complex hormonal regulation, heightened sensitivity, and limited detoxification capacity. Hence, DART plays a crucial role in assessing potential adverse effects of substances on prenatal, postnatal and juvenile development, as well as on reproductive health and fertility. Currently, most DART evaluations rely on *in vivo* models following major regulatory frameworks and international guidelines (International Council for Harmonisation, 2020; European Chemicals Agency, 2006; U.S. Environmental Protection Agency, 2025; OECD, 2018).

While *in vivo* models may be scientifically valid for testing the impact on animals and the environment, their relevance to human biology is often limited. This disconnect can result in both missed opportunities, such as deselecting compounds due to false positives in animal testing, as well as unwanted human exposures to substances inaccurately deemed safe (Beilmann et al., 2025; Greek et al., 2012; Swaters et al., 2022; Bracken, 2009; Henderson et al., 2015; Leist and Hartung, 2013).

**Fig. 1:**
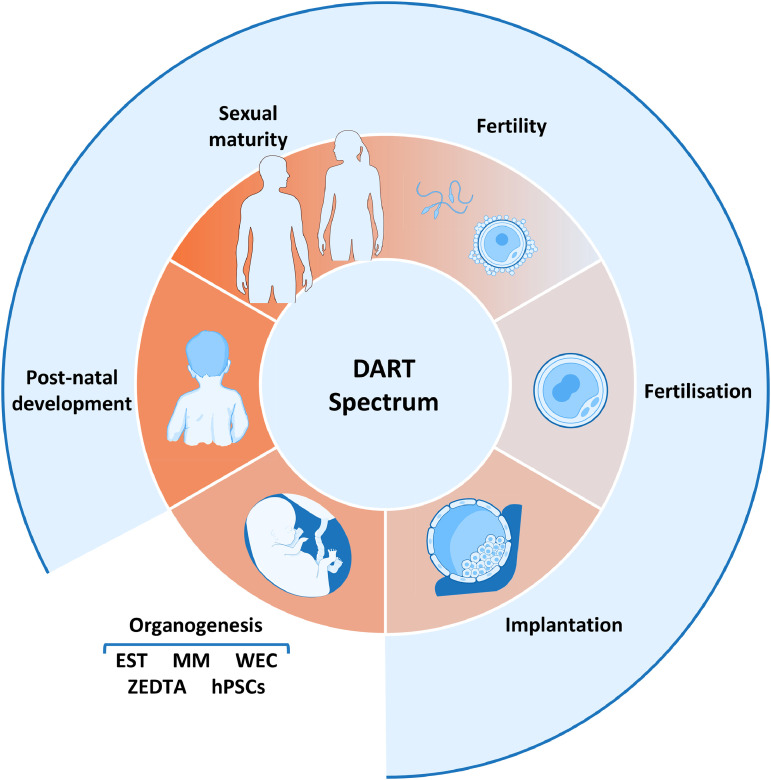
Overview of reproductive and developmental events covered by DART. Validated or widely adopted *in vitro* NAMs are shown under organogenesis: EST, embryonic stem cell test; MM, micromass assay; WEC, whole embryo culture; ZEDTA, zebrafish embryo developmental toxicity assay; hPSCs, human pluripotent stem cells. The blue-shaded outer area of the circle corresponds to areas of DART where validated *in vitro* NAMs are lacking. Icons used in this figure were primarily sourced from the NIH BioArt collection (https://bioart.niaid.nih.gov), which are in the public domain. The foetus icon was adapted from Servier Medical Art (https://smart.servier.com), licensed under Creative Commons Attribution 4.0 International (CC BY 4.0).

Within DART, several *in vitro* models have been proposed, validated or are already in use for assessing developmental toxicity, particularly in the context of embryonic and foetal development (Niethammer et al., 2022; Burbank et al., 2023; el Azhar and Sonnen, 2021; Moris et al., 2021; Mueller et al., 2025). However, two important components of DART are often overlooked or do not have yet widely accepted *in vitro* models: gonadal toxicity and placental toxicity (Fig. 1) (Niethammer et al., 2022). This gap is exemplified by the ICH S5(R3) guideline on “Detection of Reproductive and Developmental Toxicity for Human Pharmaceuticals”, which provides guidance on how data generated from NAMs can be used to support hazard identification and risk assessment in DART, but focuses exclusively on embryo-foetal development (International Council for Harmonisation, 2020).

## Assessment of gonadal and placental toxicities

Gonadal toxicity relates to the disruption of both reproductive function (gametogenesis) and endocrine processes (steroidogenesis and hormone receptor binding). The current gold standard for assessing gonadal toxicity involves a combination of repeated-dose and reproductive toxicity studies, such as OECD Test Guideline (TG) 407, 421, and 422 for chemicals (OECD, 2016; OECD, 2016; OECD, 2025) and the ICH S5(R3) guideline for pharmaceuticals (International Council for Harmonisation, 2020). These studies focus on direct effects on the gonads of rats through endpoints such as histopathological evaluation of the testes and ovaries to detect histological alterations, including spermatogenesis arrest, Leydig or Sertoli cell damage, and follicular atresia. Measurement of reproductive organ weights is performed, and sperm parameters such as count, motility, and morphology can also be assessed. In extended studies, additional endpoints such as serum levels of sex hormones may be included.

While these *in vivo* approaches provide valuable data, they are limited in their ability to reflect human-specific biology. For example, rodent spermatogenesis differs significantly from human in terms of timing, hormonal regulation, and cellular distribution, which can affect the interpretation of toxicological outcomes (Bell, 2018; Kaltsas et al., 2025; Schlegel, 2022). Similarly, ovarian follicle dynamics and steroidogenic responses vary across species, leading to potential discrepancies in sensitivity to chemical exposures (Dalbies-Tran et al., 2020; Zhao et al., 2020; Catalini and Fedder, 2020; Elvis-Offiah et al., 2022). These differences underscore the need for human-relevant *in vitro* models that better capture the nuances of gonadal function and development.

Placental toxicity, on the other hand, pertains to the placenta and its precursor structures, which play a crucial role in regulating the passage of substances from the mother to the developing foetus, as well as metabolising substances (including their activation or detoxification), and producing essential hormones. The current gold standard for assessing placental toxicity is the OECD TG 414 (OECD, 2018) for chemicals and the ICH S5(R3) guideline for pharmaceuticals (International Council for Harmonisation, 2020), which involves administering a test substance to pregnant rats and/or rabbits during the period of organogenesis. At the end of gestation, the animals are euthanised, and the uteri and foetuses are examined. The ICH S5(R3) guideline includes gross evaluation of the placenta, whereas placental weight is optional (International Council for Harmonisation, 2020). While TG 414 does not include placental assessment as a formal endpoint, the evaluation of implantation sites, resorptions, and foetal growth and malformations provides indirect insight into placental function and integrity. Placental weight and gross morphology may be recorded during foetal examinations. Although not required by the guideline, detailed placental assessments, such as histopathology or hormone production can also be included.

Placental structure and function vary significantly across mammalian species, which poses challenges for translating findings from animal models to human biology (Schmidt et al., 2021). Although rodents possess a haemochorial placenta like humans, key differences exist in trophoblast invasion depth, hormone production profiles, and gestational timelines. In humans, extravillous trophoblasts invade the maternal decidua deeply and remodel spiral arteries to ensure adequate blood flow to the foetus. In contrast, rodent trophoblast invasion is shallower and less extensive, with limited arterial remodelling (Furukawa et al., 2019; Cline et al., 2014). Structurally, the human placenta is villous, composed of branching chorionic villi, and features a haemomonochorial barrier, whereas rodents possess a labyrinthine placenta with a haemotrichorial barrier, leading to differences in permeability and transport dynamics (Hori and Kaji, 2025). Endocrine profiles also differ with human placenta becoming the major source of progesterone, whereas in rodents the corpus luteum remains the primary source of progesterone throughout pregnancy (De la et al., 2025). Comparative transcriptomic studies have revealed species-specific gene expression patterns and regulatory networks, with mouse placental development aligning only with the first half of human gestation (Soncin et al., 2018). These anatomical and molecular differences may affect the interpretation of toxicological outcomes (Strakovsky and Schantz, 2018). Human-derived *in vitro* models may offer more physiologically relevant platforms for assessing placental toxicity and improving predictive accuracy, however, since the placenta undergoes dynamic development and functional changes throughout pregnancy, different *in vitro* models may be required to reflect these developmental stages and functions.

To better understand current industry perspectives on these areas, we conducted a survey among sixteen DART experts from various sectors represented in the Health and Environmental Sciences Institute (HESI) DART committee (see Supplementary File for details). The survey was conducted anonymously and on a voluntary basis, remained open for approximately one month, and reminders were sent periodically to encourage participation. Respondents represented sectors such as pharmaceuticals, cosmetics, agrochemicals, and contract research organizations. The questionnaire included a mix of Likert-scale, multiple-choice, and open-ended questions. Some multiple-choice questions allowed multiple selections when applicable (*e.g.*, when respondents used models for different purposes), and others included an “Other” option with free-text input. The survey was beta-tested with three committee members prior to distribution to ensure clarity and relevance.

**Fig. 2:**
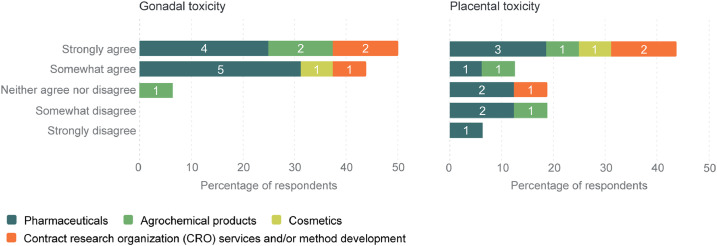
Respondents’ agreement with the statement: “Gonadal/placental toxicity is an important area of toxicity assessment in the toxicology activities of the company”. Numbers in the bars indicate the number of respondents from that industry sector. (*n* = 16).

The vast majority of respondents (94 %) identified gonadal toxicity as an important area of toxicity assessment within their organisation, while only 56 % recognised placental toxicity as a priority (Fig. 2). However, when considering only those who “strongly agreed”, the difference is less pronounced, with 50 % for gonadal toxicity and 44 % for placental toxicity.

## Industry use of and confidence in *in vivo* models in gonadal and placental toxicity assessments

**Fig. 3:**
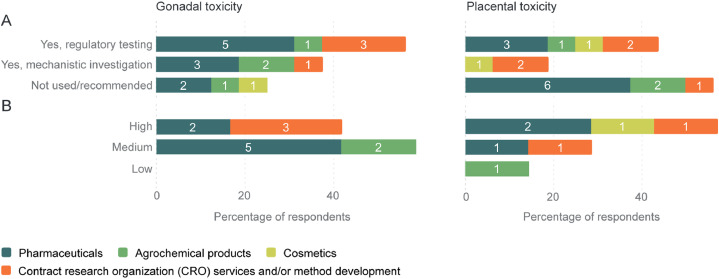
Proportion of respondents who use or recommend using *in vivo* models outside of general toxicology studies to predict human gonadal/placental toxicity (A) and their confidence in these models to conclude impact/safety (B). Numbers in the bars indicate the number of respondents from that industry sector. A – (*n* = 16); B – gonadal toxicity (*n* = 12), placental toxicity (*n* = 7).

When respondents were asked if they use or recommend *in vivo* models outside of general toxicology studies to predict human gonadal toxicity, 75 % responded positively – whether for regulatory purposes, mechanistic investigations, or both. In contrast, only 44 % indicated using or recommending *in vivo* models for assessing human placental toxicity (Fig. 3A). These findings suggest that placental toxicity remains comparatively under-addressed in DART outside of general toxicology, even if *in vivo* testing is considered. A closer look at the rationale for evaluating placental toxicity using *in vivo* models reveals that such assessments are primarily conducted for regulatory purposes, rather than for mechanistic investigations, which may contribute to its limited focus in broader toxicity assessment strategies.

Regarding the reasons for selecting specific *in vivo* models (Supplementary File, questions 13 and 14), respondents most frequently cited “regulators’ demands” and “best models available for predicting human gonadal toxicity” as the main drivers for gonadal toxicity assessments. In the case of placental toxicity, however, “regulators’ demands” were cited less often, with “best models available for predicting human placental toxicity” as the more common rationale. This further underscores the limited regulatory guidance available in this area. A similar trend was observed regarding the sources of *in vivo* models used (Supplementary File, questions 9 and 10). For gonadal toxicity, 83 % of respondents indicated that they primarily relied on regulatory guidelines, whereas for placental toxicity, only 57 % did so. In the latter case, models described in the scientific literature also represented an important source.

When asked about their level of confidence in the *in vivo* models used to evaluate potential impacts on human gonads or placenta, most respondents reported medium to high confidence (Fig. 3B). One exception was noted in the agrochemical sector, where a respondent expressed low confidence in placental toxicity models, noting that rodents were the only model used for this purpose.

## Industry perspective on and use of gonadal and placental *in vitro* NAMs

**Fig. 4:**
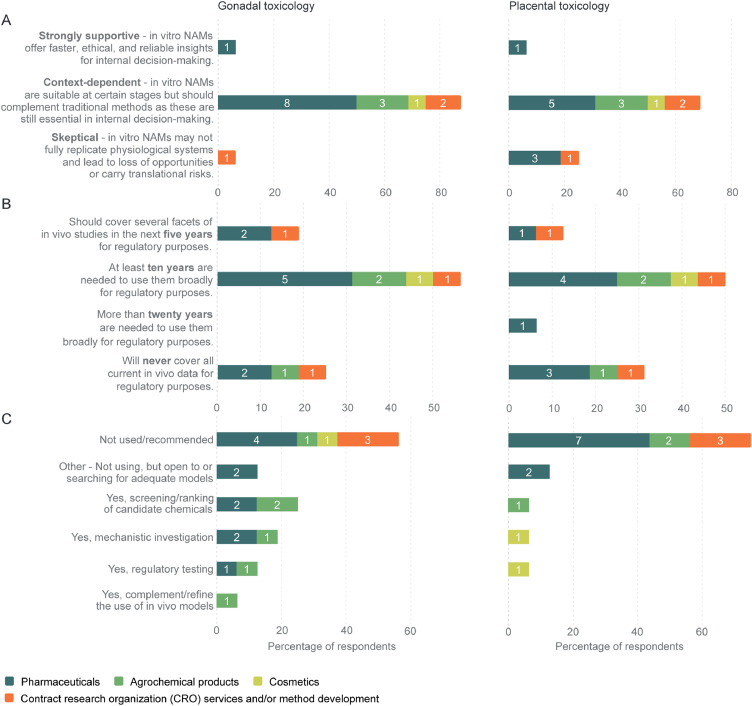
Respondents’ view on the use of *in vitro* NAMs in gonadal/placental toxicology for internal decision-making (A), their expectations regarding the timeline for the adoption of these methods for regulatory purposes (B), and current use or recommendation of these models and the context in which they are applied (C). Numbers in the bars indicate the number of respondents from that industry sector. (*n* = 16).

With respect to the use of *in vitro* NAMs for internal decision-making, respondents shared that their applicability is context-dependent. *In vitro* NAMs were considered appropriate at certain stages of decision-making, but respondents emphasise that they should complement traditional methods, which are still viewed as essential (Fig. 4A). A similar, though more cautious, perspective was expressed regarding placental toxicology. Several respondents voiced concern that *in vitro* NAMs may not fully replicate the complexity of physiological systems, potentially resulting in missed findings or translational limitations (Fig. 4A). This scepticism was also evident in respondent’s expectations for the regulatory implementation timeline of *in vitro* NAMs in placental toxicology compared to gonadal toxicology (Fig. 4B). While most respondents believed that it would take at least ten years for *in vitro* NAMs to be broadly adopted for regulatory use on placental toxicology, several anticipated it could take more than twenty years, or that current regulatory needs might never be fully met by these approaches. Notably, none of the respondents were aware of any dedicated initiatives or consortia specifically addressing NAMs in the context of gonadal or placental toxicology. However, we acknowledged the existence of broader initiatives addressing NAMs within the wider scope of DART (Fritsche et al., 2024; HESI DART Committee, 2025).

When asked whether they currently use or recommend *in vitro* NAMs for toxicity assessment, 31 % responded positively for gonadal toxicity, and only 13 % for placental toxicity (Fig. 4C). In both cases, *in vitro* NAMs were primarily applied for screening/ranking candidate chemicals (including pharmaceuticals and cosmetics), consistent with broader trends observed in investigative toxicology within the pharmaceutical industry (Pognan et al., 2023). These findings underscore the limited current application of *in vitro* NAMs in both gonadal and placental toxicity assessments.

Finally, respondents indicated two key drivers that would encourage them to adopt or recommend increased use of *in vitro* NAMs in these domains: the availability of “proven better models for predicting human toxicity” and the introduction of “new regulations requiring new animal-free models”.

## Gonadal and placental *in vitro* models

While NAMs offer the opportunity to replace animal testing, no single method is likely to capture the full range of information provided by *in vivo* studies. Therefore, “proven better models” are most likely to emerge through the combination of multiple approaches. For instance, individual NAMs may provide enough data for specific elements of an AOP, but collectively, they can contribute to a more complete AOP. The adoption of NAMs in DART is expected to follow a similar approach (Fritsche et al., 2024), *i.e.* gonadal and placental *in vitro* models can provide valuable readouts that support both screening and regulatory testing.

These *in vitro* models range from simple single-cell systems to advanced microfluidic approaches, each with distinct strengths and limitations (Table 1, Table 2, Table 3). For instance, while organoids and microfluidic systems offer greater physiological relevance by more closely mimicking *in vivo* conditions, single-cell cultures are better suited for high-throughput screening and can provide targeted mechanistic insights through relatively straightforward endpoints, though they lack the complexity to fully replicate the *in vivo* biology.

**Table 1:** *In vitro* cellular/tissue models for gametogenesis.

Gonad	2D - Primary cells	2D - Cell lines	2D – PSCs	3D – Organoids/microtissues	3D - Tissue cultures	Microfluidic and multi-organ systems	Future opportunities
**Ovary**	Oocytes are always cultured in follicles. Entire plate-attached follicles can be cultured for a more *in vivo*-like environment. **Strengths:** Human primary cells – preserved phenotype, at least for a short time. **Limitations:** Lack of 3D architecture (follicles attach to the plate and flatten) and follicle/corpus luteum environment. Low-quality oocytes starting with early follicles – short cultures if starting with late follicles. Oocytes may be hard to get for high-throughput applications. **Refs:**Malo et al., 2024 **noO noN**	No cell line is available for the study of oogenesis.	Human iPSCs differentiated into PGCLCs differentiate into oogonia. However, only mouse PGCLCs derived from iPSCs resulted in mature oocytes (although with differences from natural ones) and offspring. **Strengths:** Ability to follow the whole oogenesis *in vitro*. **Limitations:** Competent oocytes are only possible from murine. **Refs:**Hayashi et al., 2012; Yamashiro et al., 2018 **noO noN**	To study oogenesis, follicles can be cultured in scaffolds or floating allowing to keep a 3D architecture. To study folliculogenesis, few protocols are available for ovarian organoids, mainly from mixing iPSCs-derived PGCLCs/oogonia with foetal ovarian somatic cells. **Strengths:** 3D structure with all relevant ovarian cells. **Limitations:** Low throughput. **Refs:**Pors et al., 2019; Krotz et al., 2010; Chitrangi et al., 2023; McLaughlin et al., 2018; Cortvrindt and Smitz, 2002; Yang et al., 2022 **noO N**	Human tissue cultures for the study of oogenesis are not frequent. Primordial and primary follicles can be studied in tissue fragments – reserve depletion*.* After a few days in culture, developing follicles are extracted from tissue fragments and oogenesis can be studied in those. From murine, genital ridges and foetal gonads have successfully been cultured to obtain functional oocytes. **Strengths:** Germ cell-supporting cells are present from the start of culture in native architecture. **Limitations:** Low throughput. Bad quality oocytes in human cultures. **Refs:**Morohaku et al., 2016; Xiao et al., 2015 **noO N**	Mouse ovarian tissue has been used with human endometrium, fallopian tube, ectocervix and liver cultures in a microfluidic platform – female reproductive tract-on-a-chip. Good oocyte maturation achieved. **Strengths:** Assessment of interplaying organs (such as ovary and endometrium) in the same set-up. Pulsating stimulation with FSH/LH is possible. **Limitations:** Limited throughput. **Refs:**Xiao et al., 2017; Campo et al., 2023 **noO N**	A human system that can recapitulate full oogenesis from oogonium to oocyte with its supporting niche. **Limitations:** Achieving full oogenesis *in vitro* remains technically challenging due to the complexity of follicular development, niche interactions, and meiotic progression. Ethical concerns may arise from the use of embryonic or foetal tissue, or the generation of gametes *in vitro* for toxicity testing rather than fertility treatment.
**Testis**	No spermatogenesis has been achieved from spermatogonial stem cell cultures in 2D These cultures can still be useful in studying spermatogonial stem cell proliferation. **Strengths:** Simple protocols. **Limitations:** Lack of 3D architecture and reduced cell-cell interactions. **Refs:**Bashiri et al., 2023; Wu et al., 2023; Sadri-Ardekani et al., 2009 **noS noN**	Although there are several mouse cell lines representing different male germ cells, only GC-1spg (spermatogonia B - primary spermatocytes) and GC-2(spd)ts (preleptotene spermatocytes - round spermatids) are commercially available. **Strengths:** Easy genetic manipulation. High throughput. **Limitations:** Do not represent spermatogenesis or germ cell-Sertoli cell interactions. **Refs:**Verma and Parte, 2021 **noS noN**	Human iPSCs or embryonic stem cells have been differentiated into haploid germ cell-like cells. **Strengths:** Follow full spermatogenesis. Easy set-up. **Limitations:** Very limited yield and reproducibility – hard to evaluate the impact of substances tested. No morphological differentiation. Need for a pluripotent stem cell – *in vitro* spermatogenesis is not possible yet from human spermatogonia. **Refs:**Eguizabal et al., 2011; Easley et al., 2012; Gizer et al., 2024 **S noN**	Several protocols are available for testicular organoids from human testicular cells – primary and/or immortalised. No full spermatogenesis was achieved but meiotic entry has been observed. Spermatid formation in mouse testicular organoids. **Strengths:** 3D structure with all relevant testicular cells. Potential for high-throughput applications depending on the protocol. **Limitations:** 3D testicular architecture not fully recapitulated. Prepubertal tissue has greater potential for proper organisation and *in vitro* spermatogenesis but is scarce. Complex cultures, depending on the protocol. **Refs:**Wu et al., 2023; Baert et al., 2017; Sakib et al., 2019; Cui et al., 2024; Salem et al., 2023; Richer et al., 2024; Madureira Silva et al., 2025 **noS N**	Human testicular cultures show limited spermatogenesis (full for mouse and rat). **Strengths:** 3D testicular architecture is fully present with all cell types. Differentiating germ cells inside a blood-testis barrier. Easy protocols. **Limitations:** Low throughput. Tissue integrity is hard to keep throughout the culture. **Refs:**de Michele et al., 2018; Portela et al., 2019; Medrano et al., 2018; Roulet et al., 2006 **noS N**	Human testicular organoids can be placed in multi-organ chips with other organ models such as the liver. No spermatogenesis has been achieved. Mouse testicular tissue maturation and spermatogenesis for 180 days has been achieved on a microfluidic chip. **Strengths:** Prolonged support of *in vitro* spermatogenesis. Co-culture with the liver allows compound metabolisation before reaching the testis. **Limitations:** Limited throughput. Harder to assess spermatogenesis. **Refs:**Baert et al., 2020; Rajan et al., 2020; Skardal et al., 2020 **noS N**	In a microfluidic system, the addition of a vascular flow that can accurately mimic testicular access and disposal of substances, as well as a compartment representing the tubular lumen, mimicking the blood-testis barrier, is of interest. A system that would sustain both a stable spermatogonial stem cell population and germ cell differentiation would allow simultaneous read-outs for both aspects. **Limitations:** Technical complexity may affect reproducibility across labs. Ethical concerns may arise from the generation of gametes *in vitro* for toxicity testing.

FSH, follicle-stimulating hormone; LH, luteinizing hormone; PGCLCs, primordial germ cell-like cells; (i)PSCs, (induced) pluripotent stem cells. Key (related to human model): **O/S**, supports full oogenesis (O) until ovulation (metaphase II)/spermatogenesis (S); **noO/noS**, does not support full oogenesis until ovulation (metaphase II)/spermatogenesis or information about it not available; **N**, niche support is assessable; **noN**, niche support is not assessable or information about it not available.

**Table 2:** *In vitro* cellular/tissue/embryo models for gonadal steroidogenesis.

Gonad	2D – Primary cells	2D – Cell lines		2D – PSCs	3D – Organoids/microtissues	3D – Tissue cultures	Microfluidic and multi-organ systems	Fish embryo	Future opportunities
**Ovary**	Human granulosa cells – progesterone and oestradiol competent and responsive to FSH and LH. Entire plate-attached follicles can be cultured for a more *in vivo*-like environment. **Strengths:** Human primary cells – expected to follow and express all human steroidogenic pathways and machinery. **Limitations:** Granulosa cells *in vitro* differentiate into cumulus and mural cells with abnormal gene expression. Short cultures. **Refs:**Malo et al., 2024; Schipper et al., 1993; Beschta et al., 2021; Sluss et al., 1994 **G S**	NCI-H295R - human adrenocortical carcinoma cells: express all key enzymes for steroidogenesis and produce progesterone, testosterone and oestrogen. **Strengths:** Well described for testing through OECD TG456. **Limitations:** High variability in steroidogenesis depending on substrains and culture conditions. Low oestrogen and testosterone levels. No gonadal cells. **Refs:**OECD, 2022; Rao et al., 2004; Kurlbaum et al., 2020; Hecker et al., 2006; Haggard et al., 2018 **G S**	KGN, COV434 and HGL5 - human granulosa cells: variable progesterone and oestrogen production and not all steroidogenic enzymes are present. **Strengths:** Cost efficiency, easy plate assays, high throughput. **Limitations:** Variable LH and FSH responsiveness. Theca cells play an important role in androgen production that is not covered by granulosa cells alone. **Refs:**Nishi et al., 2001; Zhang et al., 2000; Rainey et al., 1994 **G S**	Human iPSCs differentiated into granulosa-like cells. Cultured as support for oocyte growth or for the regeneration of premature ovarian failure in a mouse model. Produce oestrogen when stimulated with FSH. Human ESCs differentiate into granulosa-like cells that organise into follicles. **Strengths:** Generation of patient-derived granulosa-like cells (not so relevant for high-throughput testing). **Limitations:** Human theca-like cells have not been achieved. **Refs:**Pierson Smela et al., 2023; Jung et al., 2017; Zhang et al., 2024; Lipskind et al., 2018; Cai et al., 2016 **G S**	Few protocols are available for ovarian organoids (one protocol is based on ovarian cancer tissue). No progesterone or oestrogen production was assessed. Follicles can also be cultured straight in matrix scaffolds where oestrogen and progesterone have been detected. **Strengths:** 3D structure with all relevant ovarian cells. **Limitations:** Simple protocols not available – low throughput. Granulosa overgrowth and suboptimal *in vitro* differentiation generate changes in the follicle 3D structure which might lead to disruption of follicle activities/functions and misinterpretation of results. **Refs:**Pors et al., 2019; Krotz et al., 2010; Chitrangi et al., 2023; Xiao et al., 2015 **noG noS**	Human ovarian tissue cultures have not been assessed regarding progesterone or oestrogen production. **Strengths:** 3D organ architecture is fully present. **Limitations:** Low throughput. **Refs:**Barbato et al., 2023; Del Valle et al., 2022 **noG noS**	Mouse ovarian tissue has been used with human endometrium, fallopian tube, ectocervix and liver cultures in a microfluidic platform – oestrogen and progesterone production were higher in microfluidic *versus* static cultures and responded to hCG. In another example, isolated human theca and granulosa cells were immortalised and cultured in parallel with an endometrial chamber. **Strengths:** Assessment of interplaying organs (such as ovary and endometrium) in the same set-up. Pulsating stimulation with FSH/LH is possible. **Limitations:** Limited throughput. Need for well-established organ models before microfluidic system implementation. **Refs:**Xiao et al., 2017; Fedi et al., 2023; Marrella et al., 2021 **G S**	Whole teleost fish embryos have been observed to synthesise and metabolise steroid hormones. Detection methodologies include high performance liquid chromatography, gas chromatography mass spectrometry, and radioimmuno- assays. **Strengths:** Whole organism. Smaller or larger fish allow for shorter or longer embryo cultures. **Limitations:** Contamination from maternal steroid hormones. Synthesis and metabolism of steroid hormones is most often not gonad centred. Steroidogenesis mechanisms differ from human-specific ones. **Refs:**Khan et al., 1997; Rajakumar and Senthilkumaran, 2020; Baroiller et al., 1999; Petkam et al., 2003; Yeoh et al., 1996	Multi-organ systems may include a hypothalamic-pituitary-gonadal axis in addition to the reproductive tract compartments. A cyclic hormonal system to mimic the human menstrual cycle may be useful. **Limitations:** These systems are technically complex. Standardisation and sourcing of human tissues across labs is challenging.
**Testis**	Human Leydig cells – testosterone competent in first days in culture and responsive to LH/hCG stimulation. **Strengths:** Human primary cells – expected to follow and express all human steroidogenic pathways and machinery. **Limitations:** Lack of 3D architecture and testicular environment. **Refs:**Bilinska et al., 2009; Sivakumar et al., 2006; Lejeune et al., 1998 **G S**		MA-10, BLTK1 and TM3 - mouse Leydig cells: suited for early steps of steroidogenesis, but low testosterone production. **Strengths:** Leydig cells with most steroidogenic enzymes. High throughput. **Limitations:** Do not represent accurately human steroidogenic pathways. **Refs:**Engeli et al., 2018; Forgacs et al., 2012 **G S**	Human iPSCs and ESCs differentiated into Leydig cell-like cells. Produce testosterone and express several adult Leydig cell markers and steroidogenic enzymes. **Strengths:** Generation of patient-derived Leydig cell-like cells (less relevant for high-throughput testing). **Limitations:** Testosterone levels and steroidogenic enzymes expression can vary across replicates. No response to gonadotrophins. **Refs:**Ishida et al., 2021; Shin et al., 2021; Yang et al., 2020; Chen et al., 2019; Li et al., 2019; Sato et al., 2025 **nG S**	Several protocols are available for testicular organoids from human testicular cells – primary and/or immortalised. Testosterone competent with variable LH/hCG responsiveness. **Strengths:** 3D structure with all relevant testicular cells. Potential for high-throughput applications. **Limitations:** 3D testicular architecture not recapitulated – prepubertal tissue has greater potential but is scarce. **Refs:**Baert et al., 2017; Madureira Silva et al., 2025, Sakib et al., 2019, Cui et al., 2024; Pendergraft et al., 2017 **G S**	Human testicular cultures produce testosterone – prepubertal ones show higher levels over longer periods. **Strengths:** 3D testicular architecture is fully present with all cell types. **Limitations:** Low throughput. Prepubertal testicular tissue is scarce. **Refs:**de Michele et al., 2018; Portela et al., 2019; Medrano et al., 2018; Roulet et al., 2006 **G S**	Testicular organoids can be placed in chips with other organ compartments. In a testis-liver multi-organ chip, testosterone was detected and metabolised by the liver compartment. Mouse tissue cultured for 120 days in a microfluidic device showed testosterone competence when stimulated with LH. Human tissue has also been placed in a chip resulting in increased viability. **Strengths:** Possibility to metabolise compounds before reaching the testis or study systemic toxicity *vs.* endocrine disruption. Long-term, repeated dose testing. **Limitations:** Limited throughput, harder to establish and more complex to study/read endpoints. **Refs:**Baert et al., 2020; Rajan et al., 2020; Skardal et al., 2020; Komeya et al., 2016; Sharma et al., 2022 **G S**		Transgender testicular tissue is abundant, contrary to other human sources, making it useful in high-throughput organoid generation (Madureira Silva et al., 2025). Multi-organ systems show potential for the study of the hypothalamic-pituitary-gonadal axis or other relevant organ interactions with the testis. **Limitations:** Primary tissue variations may affect reproducibility. Standardisation across studies is challenging.

FSH, follicle-stimulating hormone; LH, luteinizing hormone; hCG, human chorionic gonadotrophin; (i)PSCs, (induced) pluripotent stem cells; ESCs, embryonic stem cells. Key (related to human model): **G**, responds to gonadotrophins; **noG**, does not respond to gonadotrophins or information about it unavailable; **S**, steroidogenesis is present; **noS**, steroidogenesis is not present or information about it is unavailable.

**Table 3:** *In vitro* cellular/tissue models for placental function.

2D – Primary cells	2D – Cell lines	2D – PSCs	3D – Organoids/microtissues	3D – Tissue cultures	Microfluidic and multi-organ systems	Future opportunities
Human trophoblast cells can be isolated from term or early pregnancy placenta. Produce both a hCG and hPL. **Limitations:** Human trophoblast cells have limited proliferative ability and fail to form complete monolayers. 2D set-up is not compatible with two compartments. **Refs:**Li et al., 2023 **H noB D**	There are several cell lines from choriocarcinoma such as BeWo, JEG-3, and JAR. **Strengths:** Easy to keep and widely used. **Limitations:** Exhibit high heterogeneity and very different DNA methylation pattern from primary trophoblasts. **Refs:**Li et al., 2023 **H B D**	Trophoblast stem cells can be derived from iPSCs and embryonic stem cell lines and further specify into syncytiotrophoblasts and extravillous trophoblasts. **Strengths:** Multipotency of trophoblast stem cells. Can be kept for >30 passages. **Limitations:** When used in 2D, applicability is reduced. **Refs:**Li et al., 2023; Tietze et al., 2024; Jang et al., 2022; Okae et al., 2018; Takahashi et al., 2007; Bai et al., 2021 **H B D**	Placental (trophoblast) organoids have been derived from first-trimester cytotrophoblasts resulting in highly differentiated *in vivo*-like trophoblasts with syncytiotrophoblasts and extravillous trophoblasts. Trophoblast organoids produce hCG and show barrier integrity. **Strengths:** Miniaturised cultures that can be kept for longer than explants. Cultures have been kept for longer than a year. **Limitations:** Use of Matrigel in several protocols. Syncytiotrophoblasts towards the organoid cavity while cytotrophoblasts face the matrix – not *in vivo*-like. First-trimester placental tissue may be hard to obtain. **Refs:**Li et al., 2023; Hori et al., 2024; Haider et al., 2018; Sheridan et al., 2020; Turco et al., 2018 **H B D**	Placenta *ex vivo* and placenta explants cultures are performed for a few hours to 3 days. hCG is produced. Possible to test the passage of substances from mother to foetus and the ability of the placenta to retain and metabolise substances in an *in vivo*-like structure. Human amniotic membrane biopsies can be cultured for up to 21 days. **Strengths:** Human membranes and placental 3D structures. **Limitations:** Short placental cultures, hard to establish and keep, and low rate of successful experiments. **Refs:**Li et al., 2023; Poženel et al., 2019; Luconi et al., 2022; Miller and Loch-Caruso, 2010; Watson et al., 1995 **H B D**	Placenta explant cultures in a microfluidic set-up have their lifetime extended. iPSCs-derived trophoblasts cultured in a microfluidic 3D environment show better placental phenotype. In amnion epithelial with decidual cell cultures, the controlled environment of a chip allows physical and fluidic isolation between foetal and maternal compartments and better transfer of biochemicals between compartments compared to transwell cultures. **Strengths:** Shear stress can be replicated. Clear different chambers (maternal/foetal) can be achieved. In the case of iPSCs-derived trophoblasts, high throughput can be achieved. **Limitations:** Although placenta explant culture time is extended in a microfluidic set-up, was still limited to two weeks and lost its intact structure. In the case of iPSCs-derived trophoblasts, the culture is still missing all other important cell types in the placental barrier such as endothelial or immune cells and fibroblasts. **Refs:**Li et al., 2023; Mai et al., 2022; Richardson et al., 2020; Lermant et al., 2023; Pemathilaka et al., 2019; Lee et al., 2016; Blundell et al., 2018 **H B D**	Implement trophoblast organoids in a microfluidic system with developmental organ models. Incorporate into trophoblast organoids a vascular network that more accurately mimics the placenta. **Limitations:** Limited availability of first-trimester samples may pose logistical challenges across labs.

hCG, human chorionic gonadotrophin; hPL, human placental lactogen; (i)PSCs, (induced) pluripotent stem cells. Key (related to human model): **H**, hormone production is present, at least hCG (and hPL, progesterone, oestrogen when tested); **B**, allows barrier function studies; **noB**, barrier function studies are challenging to establish; **D**, allows trophoblast differentiation studies.

To compile the models summarised in Table 1, Table 2, Table 3, we conducted a narrative literature review using PubMed. Search terms included combinations of keywords related to the organ of interest and model type, such as: placenta, testis, ovary, spermatogenesis, oogenesis, steroidogenesis, testosterone, oestrogen, trophoblast cells, Leydig cells, granulosa cells, primary cell cultures, cell lines, pluripotent stem cells, organoid, tissue cultures, explant cultures, on-a-chip, microfluidic system, and fish embryo. To ensure comprehensive coverage, we additionally used AI-assisted literature exploration tools (*i.e.*, Copilot and ChatGPT) to identify potentially relevant articles that may have been missed in PubMed searches. We included peer-reviewed studies describing human *in vitro* models, or non-human models only when no equivalent human model was available, with a focus on the most advanced achievements in modelling gonadal or placental biology. Later studies that did not provide additional novelty or advancement in biological modelling compared with earlier work were excluded.

To assess industry’s perspectives on *in vitro* models for gametogenesis, gonadal steroidogenesis, and placental function, survey participants were asked to rate their acquaintance with and confidence in these models. Acquaintance was defined as having used or reviewed data generated from the model, while confidence referred to the perceived reliability of the model for safety translation.

**Fig. 5:**
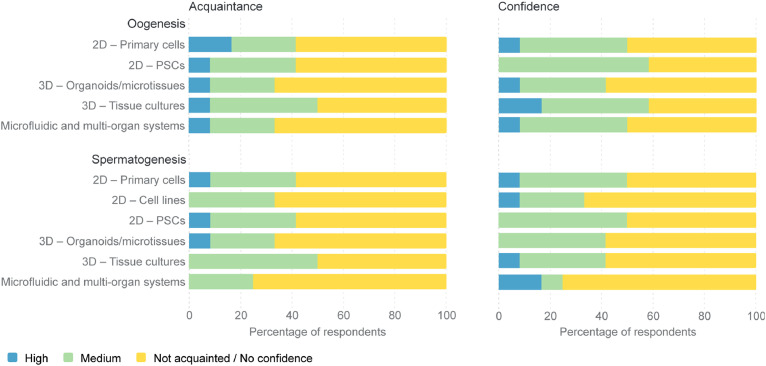
Respondents’ self-reported level of acquaintance with and confidence in various *in vitro* models for the study of oogenesis and spermatogenesis. (*n* = 12).

**Fig. 6:**
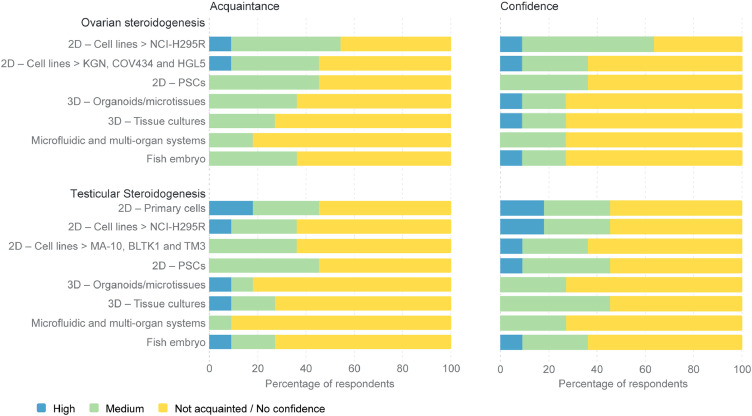
Respondents’ self-reported level of acquaintance with and confidence in various *in vitro* models for the study of ovarian and testicular steroidogenesis. (*n* = 11).

**Fig. 7:**
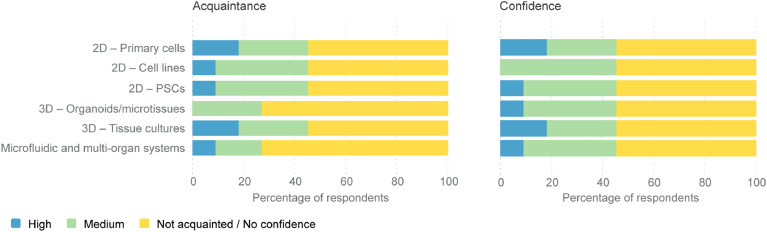
Respondents’ self-reported level of acquaintance with and confidence in various *in vitro* models for the study of placental function. (*n* = 11).

Overall, respondents reported either no familiarity or only superficial acquaintance with most models (Figs. 5, 6, and 7). A notable exception was a respondent from the cosmetics industry, who indicated higher levels of both acquaintance and confidence across several *in vitro* methods, possibly reflecting the sector’s longstanding reliance on animal-free regulatory testing strategies. Interestingly, respondents tended to rate their confidence in models similarly to their level of acquaintance, with deviations of no more than one level (*e.g.*, high/medium or medium/none). This pattern was particularly consistent among those not acquainted with a model, who almost always rated no confidence – except for four respondents who occasionally selected medium confidence despite no prior acquaintance.

## *In vitro* cellular and tissue models for gametogenesis

Gametogenesis – oogenesis in females and spermatogenesis in males – is the process by which diploid germ stem cells differentiate into haploid gametes. This complex process, including meiosis, relies on a supporting cell niche, is highly regulated, and differentially active depending on the sex and developmental stage of the individual, making it difficult to recapitulate *in vitro*. Despite these challenges, several *in vitro* cellular/tissue models have been proposed in the literature, covering different aspects of gametogenesis with potential for use in toxicity assessments (Table 1). While both oogenesis and spermatogenesis have been successfully modelled *in vitro* using rodent systems – pluripotent stem cells (PSC) for oogenesis (Hayashi et al., 2012; Morohaku et al., 2016) and tissue cultures for spermatogenesis (Matsumura et al., 2023; Sato et al., 2011) – human *in vitro* models have so far been limited to spermatogenesis (Eguizabal et al., 2011; Easley et al., 2012; de Michele et al., 2018). However, all of these models remain inefficient in terms of haploid germ cell yield and lack a supporting cell niche in embryonic and induced PSCs-derived systems.

Survey respondents were most acquainted with 2D cultures of primary cells and tissue-based models for oogenesis, with the latter receiving the highest confidence levels (Fig. 5). In contrast, microtissues and multi-organ systems were least known, and microtissues were associated with the lowest confidence.

For spermatogenesis, primary cell cultures, PSC-based systems, and tissue cultures were the most familiar (Fig. 5). Primary cell cultures also received the highest confidence ratings. Multi-organ systems were the least known, and along with cell line-based and organoid cultures, were rated with the lowest levels of confidence.

## *In vitro* cellular, tissue and embryonic models for steroidogenesis

Steroidogenesis is the biochemical process by which cholesterol is converted into steroid hormones. Although this process occurs in multiple organs and involves various cell types, gonadal steroidogenesis specifically focuses on the production of sex steroid hormones, with different roles in the testis and ovary (Miller and Auchus, 2011). In the adult testis, Leydig cells primarily synthesise testosterone, whereas in the adult ovary, granulosa and theca cells are responsible for producing oestradiol and progesterone. However, the behaviour and function of Leydig, theca, and granulosa cells vary significantly across developmental stages. For instance, while theca and granulosa cells are not steroidogenically active until puberty, an earlier population of Leydig cells, known as foetal Leydig cells, plays a crucial role in maintaining physiological levels of testosterone during male foetal development. These foetal Leydig cells do not produce the enzyme HSD17B3, which converts androstenedione into testosterone. Instead, immature Sertoli cells producing HSD17B3 are required to complete this conversion (Planinic et al., 2024). Therefore, to accurately assess the impact of toxicants on human foetal masculinisation or early postnatal testosterone surges, rather than on pubertal or adult stages, *in vitro* models must include both foetal Leydig cells and immature Sertoli cells. Accurate modelling of human gonadal steroidogenesis, including sex-specific and developmental stage differences, is essential for human safety assessments.

*In vitro* models used in steroidogenesis studies may consist solely of steroidogenesis-competent cell types or incorporate more complex systems such as organoid and tissue cultures involving multiple cell types, or even whole-organism models like fish embryos (Table 2). Some human models, particularly those derived from prepubertal tissue or from progenitor or pluripotent stem cells, may better reflect early developmental physiology. However, most published models do not explicitly characterise the steroidogenically active cells in terms of their developmental stage, which limits their applicability for assessing life stage-specific toxicological effects.

Currently, the human adrenocortical carcinoma cell line H295R serves as the standard *in vitro* model for assessing steroidogenesis, as described in OECD TG 456 (OECD, 2022). These cells are capable of producing progesterone, oestrogen, and testosterone, and respond to gonadotrophin stimulation (ChV et al., 2004). However, hormone production by these cells is highly susceptible to culture conditions and levels of testosterone and oestrogen are generally low (Kurlbaum et al., 2020; Hecker et al., 2006; Strajhar et al., 2017). Additionally, studies have shown that rat Leydig cells exhibit higher sensitivity to chemical-induced changes in testosterone production; effects that are not detected in H295R cells (Botteri Principato et al., 2018). This indicates that different steroidogenic cell types may respond variably to chemical exposure, underscoring the need for model diversity.

For ovarian and testicular steroidogenesis, respondents were most acquainted with primary granulosa and Leydig cell cultures and expressed the highest confidence in these models, along with the H295R cell model, despite its shortcomings (Fig. 6). On the other hand, microfluidic and multi-organ systems were the least known and received the lowest confidence ratings for assessing steroidogenesis.

## *In vitro* cellular and tissue assays for placental function

As previously discussed, the placenta displays considerable anatomical diversity across mammalian species. In humans, the placenta is a transient, maturing organ present during pregnancy/gestation that develops from embryo trophoblast cells, which populate the maternal endometrium during implantation and subsequently differentiate into specialised subtypes (Cindrova-Davies and Sferruzzi-Perri, 2022). The placenta’s continuous maturation during gestation, combined with its multi-structural architecture comprising both maternal and embryonic/foetal contributions, makes it a challenging organ to model *in vitro*. Key placental functions include hormone production, barrier function, and metabolic activity. Thus, the inclusion of these functions and the ability to study the trophoblast differentiation potential are key for a placenta model (Table 3).

The models with which the respondents were most acquainted and confident with were primary cell and tissue cultures (Fig. 7). In contrast, respondents were least acquainted with organoids and had the least confidence in cell lines.

## Challenges and outlook

In recent years, there has been an increasing push towards animal-free methods, with *in silico* and *in vitro* methods gradually taking more ground in research and development activities. However, this momentum has yet to translate into a more substantial shift within the field of regulatory toxicology. Progress is uneven due to varying legislative requirements and differences across toxicological subdisciplines. While certain *in vitro* models for developmental toxicity are increasingly being used, primarily for screening purposes rather than regulatory applications (Mueller et al., 2025; Fritsche et al., 2024), this trend is not observed for gonadal and placental toxicology, which continue to rely heavily on animal models.

Currently, a single *in vivo* DART study can involve hundreds to thousands of animals, depending on the guideline followed, with OECD TG 443 being among the most animal-intensive ones (OECD, 2018). The results of our survey show that industry remains largely dependent on animal models to comply with regulatory demands, even when doubts persist regarding the predictive value of animal data for human outcomes. Among NAMs, *in vitro* models for steroidogenesis are emerging as particularly relevant, given their increasing use in regulatory contexts, especially in light of recent regulatory developments that classify endocrine disruption as a distinct hazard class under the European Classification, Labelling and Packaging regulation (European Chemicals Agency, 2025).

Although no single NAM can yet replicate the full breadth of data obtained from an animal model, combining several NAMs (integrated testing strategy) can generate more human-relevant insights (Rovida et al., 2015). However, our survey reveals that industry professionals are generally unfamiliar with many *in vitro* models for gonadal and placental toxicology. This lack of acquaintance directly impacts confidence as, simply put, one cannot trust what one does not know. Additionally, the current lack of robust and relevant human NAM data likely contributes to this lack of confidence. Despite this, the majority of respondents showed an optimistic view toward the integration of gonadal and placental *in vitro* NAMs into both internal decision-making and for regulatory purposes. Notably, one respondent reported plans to use a placenta-on-a-chip model currently under development.

It is important to acknowledge that the number of survey respondents was limited (*n* ≤ 16), with variable response rates across questions. Additionally, the industrial chemicals sector was not represented due to a lack of participation of respondents in this area. While the survey provides valuable insights, its exploratory nature and limited sample size present inherent limitations, including potential response bias and sectoral imbalance. Descriptive statistics were used to summarise responses, and exploratory analyses were performed to identify potential relationships between industry sector and responses, as well as between levels of acquaintance and confidence. However, due to the small and uneven sample, no statistically robust patterns could be confirmed. Nevertheless, participants were DART experts actively engaged in the challenges of developmental and reproductive toxicity assessment, providing meaningful insights.

While survey respondents expressed optimism regarding the integration of *in vitro* NAMs into regulatory frameworks within the next 10 to 20 years, particularly in gonadal toxicity, this timeline must be critically assessed considering current regulatory initiatives. FDA’s “Roadmap to Reducing Animal Testing in Preclinical Safety Studies” (U.S. Food and Drug Administration, 2025), alongside EMA’s guideline on the regulatory acceptance of NAMs (European Medicines Agency, 2016), and the European Commission’s ongoing roadmap efforts “Towards Phasing Out Animal Testing for Chemical Safety Assessments” (Cronin et al., 2025; European Commission, 2025; European Commission, 2025), represent important steps forward. However, their implementation is still in early phases, may vary across sectors, and most importantly, it depends on regulatory acceptance. The need for robust validation data and sector-specific requirements may progress slowly. Moreover, the lack of harmonised global standards and limited familiarity among risk assessors further complicate adoption. Therefore, while the 10 to 20-year horizon is plausible for certain applications, widespread regulatory integration of NAMs, particularly in underrepresented areas like gonadal and placental toxicity, may require sustained investment, coordinated efforts, and policy reform to become a reality.

Outside the European Union and United States regulatory landscapes, it is important to acknowledge that regulatory acceptance of NAMs varies globally. Countries such as Japan, Canada, South Korea, and India are also advancing alternative testing strategies, though at different paces and with varying levels of regulatory integration (Government of Canada, 2025; Bhuller et al., 2021; National Institute of Food and Drug Safety Evaluation, 2023; Government of Canada, 2023; Yamazaki and Ishida, 2025; Kojima, 2025; Parvatam, 2025). In the southern hemisphere, Brazil passed a landmark ban on animal testing for cosmetics in July 2025 (National Congress of Brazil, 2025), aligning itself with other close countries such as Ecuador, Colombia and Chile (El Congresso de Colombia, 2020; Republica del Ecuador - Asamblea Nacional, 2017; Cámara de Diputados Chile, 2020) in cruelty-free standards and reinforcing the urgency of adopting validated non-animal methods. New Zealand and Australia have banned new animal test data for cosmetics since 2015 and 2020, respectively (Australian Government Department of Health D and A, 2025; New Zealand Parliament, 2015), and Australia is now integrating NAMs into risk assessment strategies, particularly in environmental and food safety contexts (Australian Government Department of Health D and A, 2022).

The OECD plays a key role in promoting international harmonisation through the development of test guidelines and guidance documents that support the use of NAMs across jurisdictions (More Knowledge with Fewer Animals Project, 2025; OECD, 2024). However, differences in legal frameworks, validation requirements, and institutional readiness can represent challenges for global alignment. Greater international collaboration and mutual recognition of validated NAMs will be essential to ensure their broader adoption and to facilitate consistent safety assessments across regions. The EMA’s guideline on the regulatory acceptance of 3Rs approaches outlines essential criteria such as biological relevance, reproducibility, and inter-laboratory robustness (European Medicines Agency, 2016). A notable example of successful validation is the OECD Test Guideline 456, which formalised the H295R steroidogenesis assay for endocrine disruption screening (OECD, 2022).

Based on this literature review and survey, the following actions could be proposed to advance the use of *in vitro* NAMs in gonadal and placental toxicity assessment:

• Invest in research to improve the qualification and reliability of new and existing gonadal and placental *in vitro* models, improve human relevant readouts translation, and standardise protocols.
• Raise familiarity of industry professionals with gonadal and placental *in vitro* models, by highlighting their strengths, limitations, and strategic opportunities for implementation.
• Establish and support initiatives and consortia that bring together stakeholders from industry, academia, and regulatory bodies, fostering innovation and acceptance of gonadal and placental *in vitro* NAMs.
• Advocate for regulatory changes that encourage the gradual adoption of NAMs where possible and reduce the reliance on *in vivo* models in DART, including underexplored areas such as gonadal and placental toxicology.

Building on these proposed actions, several concrete steps can further support the integration of *in vitro* NAMs into regulatory toxicology. Public-private partnerships, such as those facilitated by HESI, or the initiatives “Animal-free Safety assessment of chemicals: Project cluster for Implementation of novel Strategie” – ASPIS and “Partnership for the Assessment of Risks from Chemicals” – PARC, can play a pivotal role in fostering collaborative model development and validation. Targeted funding mechanisms are essential to generate robust human-relevant data and to standardize protocols across laboratories, ensuring reproducibility and regulatory confidence. Additionally, pilot studies that apply NAMs in real-world regulatory submissions can help demonstrate feasibility and build trust among regulators. These efforts, combined with transparent reporting and active stakeholder engagement, will be critical to overcoming current barriers and accelerating the adoption of NAMs in safety assessment frameworks.

In addition to these strategic actions, it is important to acknowledge the practical barriers that may hinder the broader adoption of *in vitro* NAMs. Many advanced models, such as microfluidic systems and organoids, offer high physiological relevance but are often limited by low throughput, high costs, and technical complexity. Moreover, reproducibility across laboratories remains a challenge, particularly for models that rely on primary human tissues or complex differentiation protocols. These limitations can affect scalability and regulatory confidence. Addressing these issues will require investments in protocol standardization, automation, and inter-laboratory validation studies, which are essential to ensure that promising models can be reliably implemented in diverse research and regulatory settings.

In conclusion, despite advances in NAM development and increased societal and scientific interest, the fields of gonadal and placental toxicities remain insufficiently prepared for a transition away from animal models. Limited exposure to these newer *in vitro* approaches remains a key obstacle. Bridging this gap will require greater investment, the generation of high-quality human-relevant data combined with exposure modelling, increased awareness among DART risk assessors, and active collaboration across stakeholders. These efforts will be essential to enable more human-relevant and ethically responsible DART toxicity testing in the years ahead, ultimately leading to better protection of human health.

## Funding

This project was conducted with financial support from the Scientific Research Foundation-Flanders (G026223N) to YB, the Strategic Research Program 89 from the VUB to EG, and the Mireille Aerens Chair to TV.

## CRediT authorship contribution statement

**Samuel Madureira Silva:** Writing – review & editing, Writing – original draft, Project administration, Methodology, Formal analysis, Data curation, Conceptualization. **Steven Van Cruchten:** Writing – review & editing, Resources, Methodology, Data curation, Conceptualization. **Freddy Van Goethem:** Writing – review & editing, Conceptualization. **Tamara Vanhaecke:** Writing – review & editing, Funding acquisition. **Ellen Goossens:** Writing – review & editing, Supervision, Funding acquisition. **Yoni Baert:** Writing – review & editing, Supervision, Methodology, Funding acquisition, Conceptualization.

## Declaration of competing interest

The authors declare the following financial interests/personal relationships which may be considered as potential competing interests: Yoni Baert reports financial support was provided by Research Foundation Flanders. Tamara Vanhaecke reports financial support was provided by Mireille Aerens Chair. If there are other authors, they declare that they have no known competing financial interests or personal relationships that could have appeared to influence the work reported in this paper.

## Data Availability

The individual survey responses have been deposited in a publicly accessible database. The data can be accessed at: https://doi.org/10.5281/zenodo.16980666.

## Appendix Supplementary materials

A supplementary file is available with the survey details, questions and responses. Image, application 1
