# Supplementary material for: In vitro models for gonadal and placental toxicity: A review and industry survey
Source: NAM J. 2025 Sep 13;1:100052. doi: 10.1016/j.namjnl.2025.100052 (PMC13289032; doi:10.1016/j.namjnl.2025.100052)

DART experts survey - gonadal and placental toxicology

This survey was conducted by the authors of this publication and distributed to DART experts from the Health and Environmental Sciences Institute (HESI) DART committee. The aim was to clarify current industry practices and perspectives on gonadal and placental toxicology, with a particular focus on *in vitro* NAMs.

Each survey question is presented as the title of the corresponding graphical representation or table displaying the results. The number of respondents for each question is indicated by “n”. When the “other” option was selected and respondents provided relevant information, these responses are included under the “other” category in the respective graphs or tables.

1. What is the main area of activity in your company? n=16

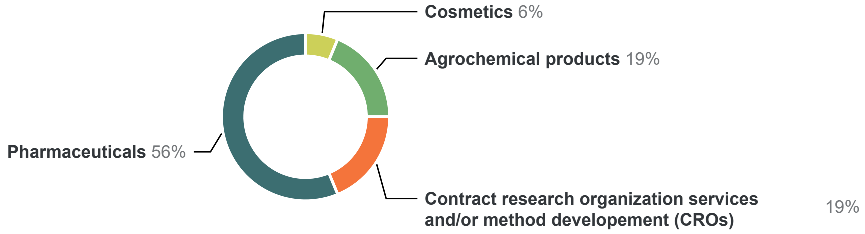

What is the main area of activity in your company? n=16

Q1 - What is the main area of activity in your company? - Selected Choice	Percentage	Count
Pharmaceuticals	56%	9
Agrochemical products	19%	3
Cosmetics	6%	1
Contract research organization (CRO) services and/or method development	19%	3

2. What type of toxicology activities are conducted? n=16

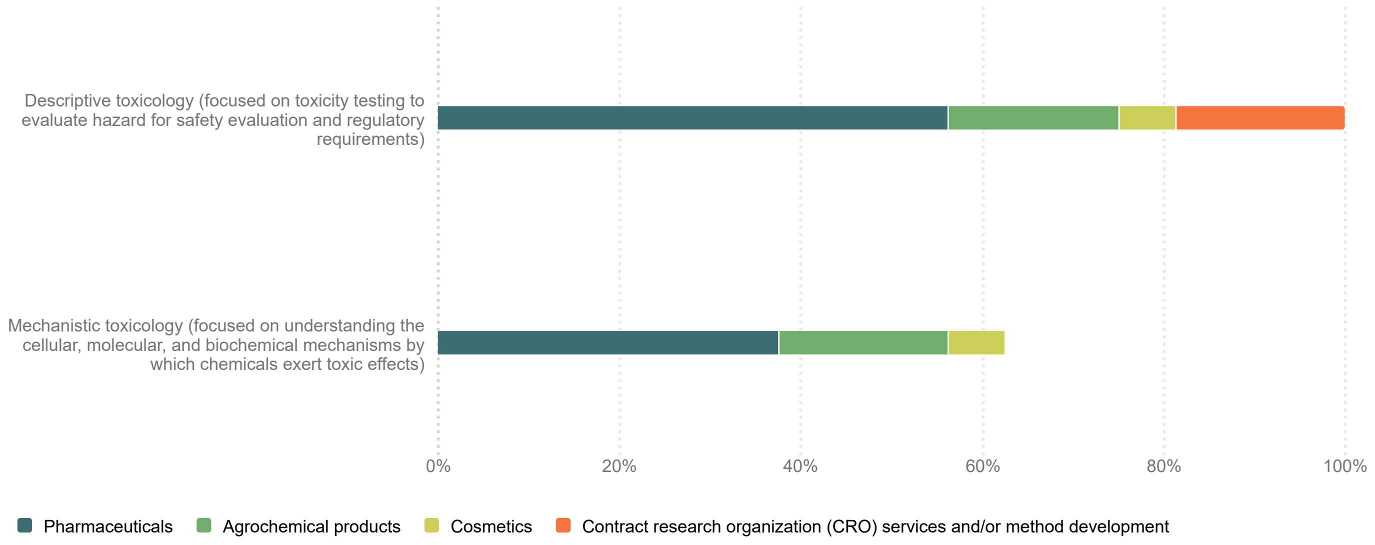

What type of toxicology activities are conducted? n=16

Q2 - What type of toxicology activities are conducted?	Percentage	Count
Mechanistic toxicology (focused on understanding the cellular, molecular, and biochemical mechanisms by which chemicals exert toxic effects)	63%	10
Descriptive toxicology (focused on toxicity testing to evaluate hazard for safety evaluation and regulatory requirements)	100%	16

3. Do you agree with the following statement? n=16
Gonadal toxicity is an important area of toxicity assessment in the toxicology activities of your company.

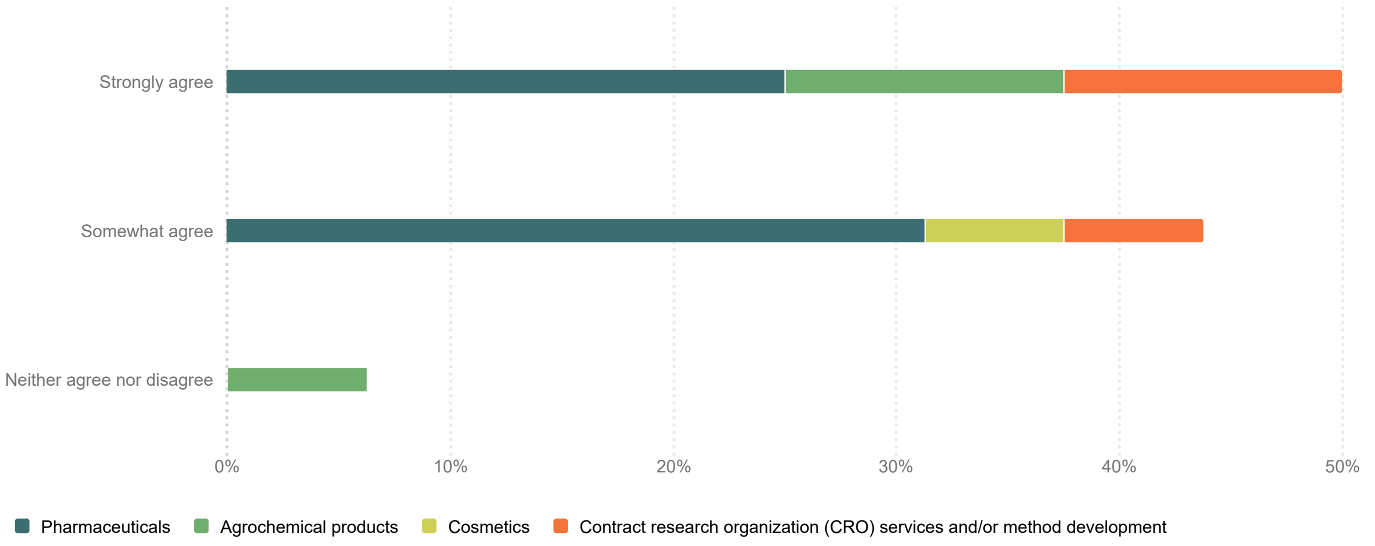

4. Do you agree with the following statement? n=16
Placental toxicity is an important area of toxicity assessment in the toxicology activities of your company.

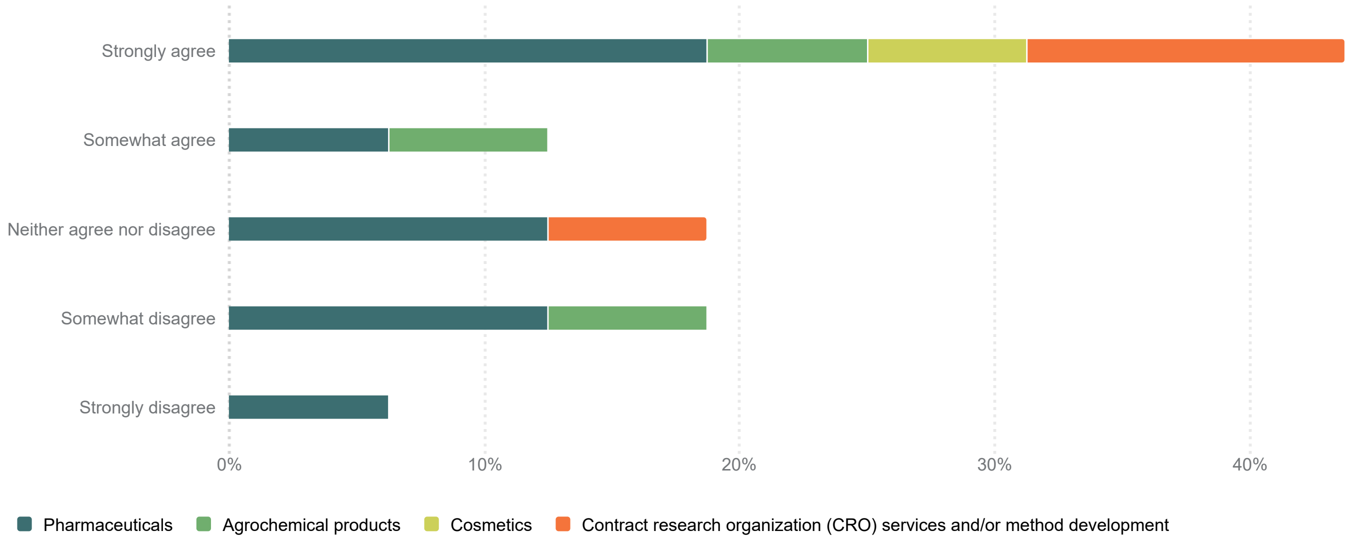

5. Do you use/recommend using in vivo models outside of general toxicology studies to predict human gonadal toxicity? n=16

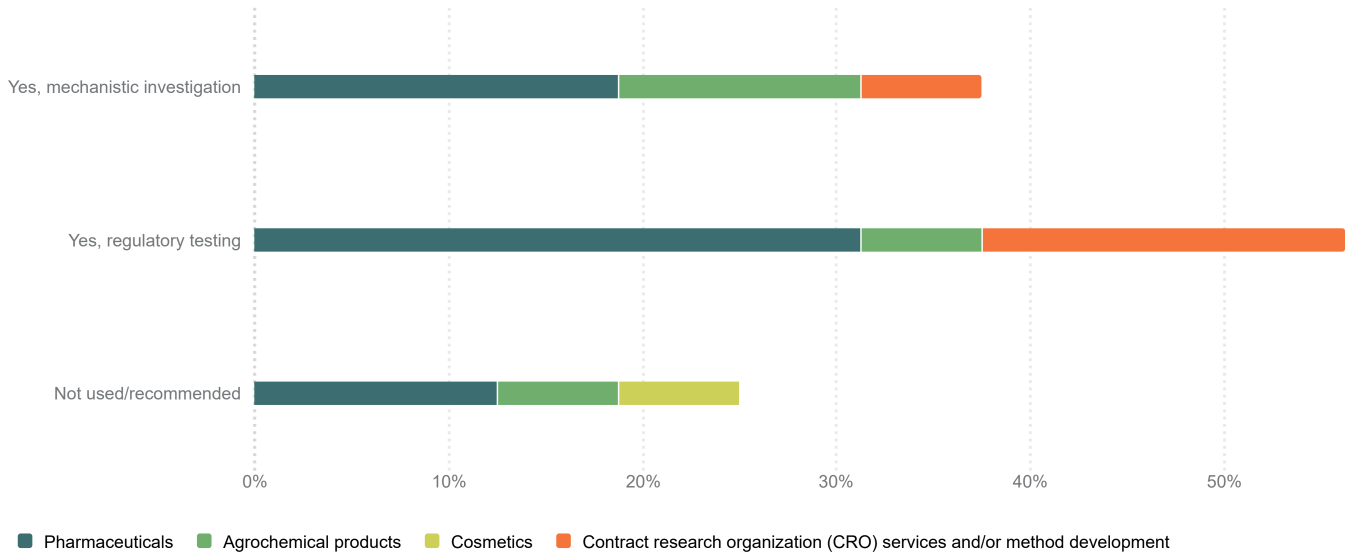

Do you use/recommend using in vivo models outside of general toxicology studies to predict human gonadal toxicity? n=16

Q4 - Do you use/recommend using in vivo models outside of general toxicology studies to predict human gonadal toxicity?	Percentage	Count
Not used/recommended	25%	4
Yes, regulatory testing	56%	9
Yes, mechanistic investigation	38%	6

6. Do you use/recommend using in vivo models outside of general toxicology studies to predict human placental toxicity? n=16

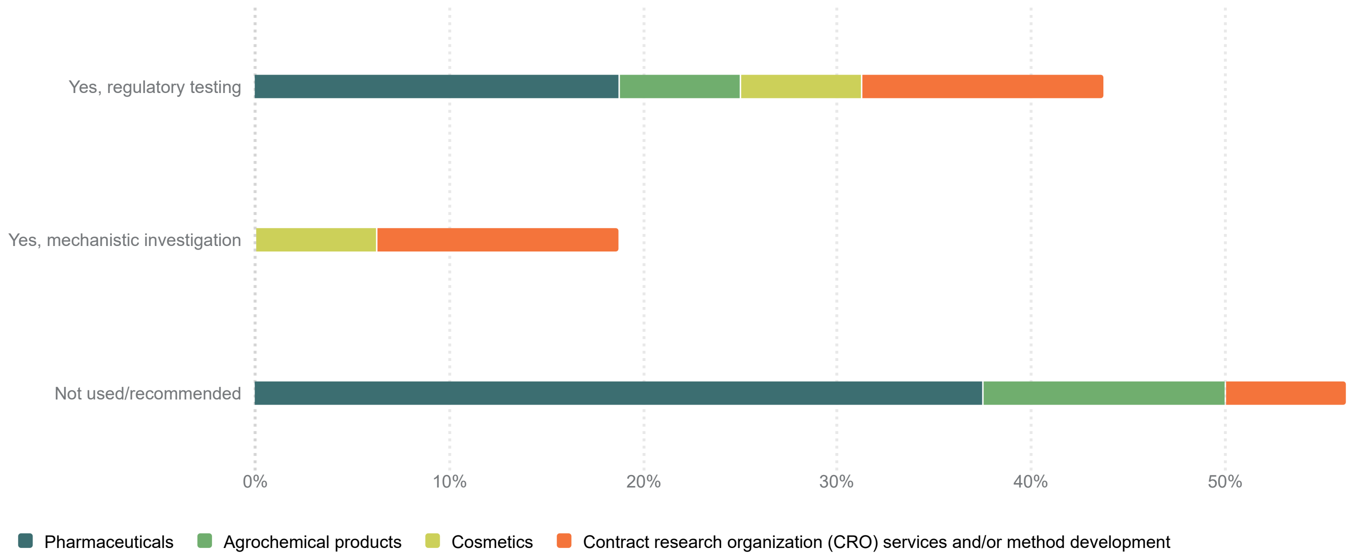

Do you use/recommend using in vivo models outside of general toxicology studies to predict human placental toxicity? n=16

Q13 - Do you use/recommend using in vivo models outside of general toxicology studies to predict human placental toxicity?	Percentage	Count
Not used/recommended	56%	9
Yes, regulatory testing	44%	7
Yes, mechanistic investigation	19%	3

7. What in vivo models do you use/recommend using to predict human gonadal toxicity? n=12

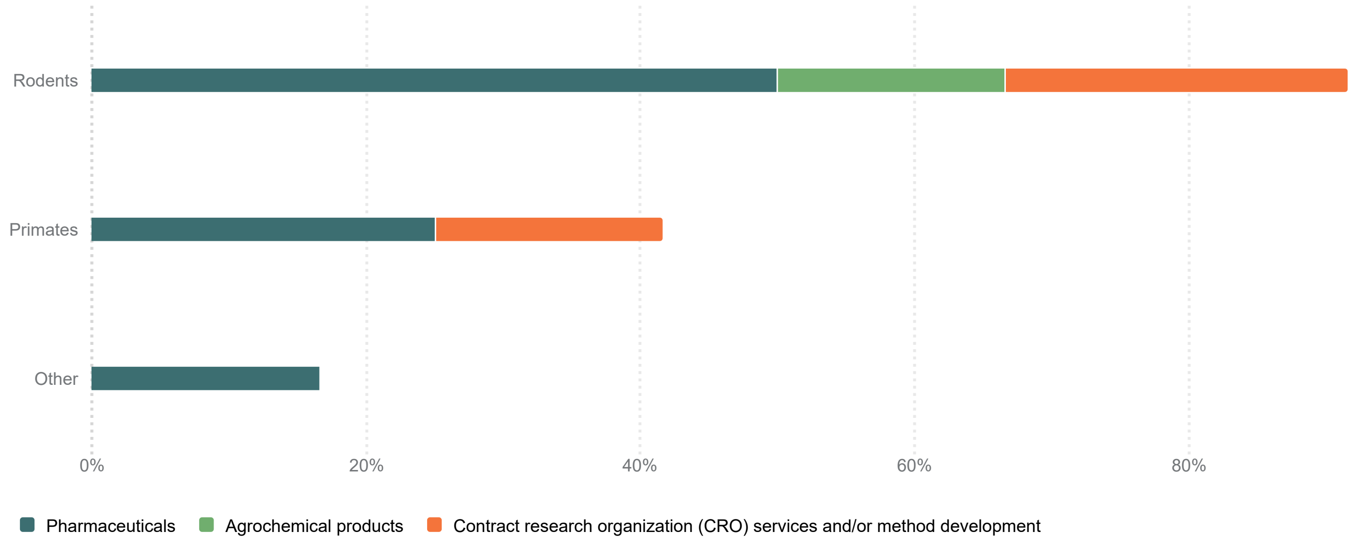

What in vivo models do you use/recommend using to predict human gonadal toxicity? n=12

Q5 - What in vivo models do you use/recommend using to predict human gonadal toxicity? - Selected Choice	Percentage	Count
Rodents	92%	11
Primates	42%	5
Other	17%	2

rabbit (rarely)

8. What in vivo models do you use/recommend using to predict human placental toxicity? n=7

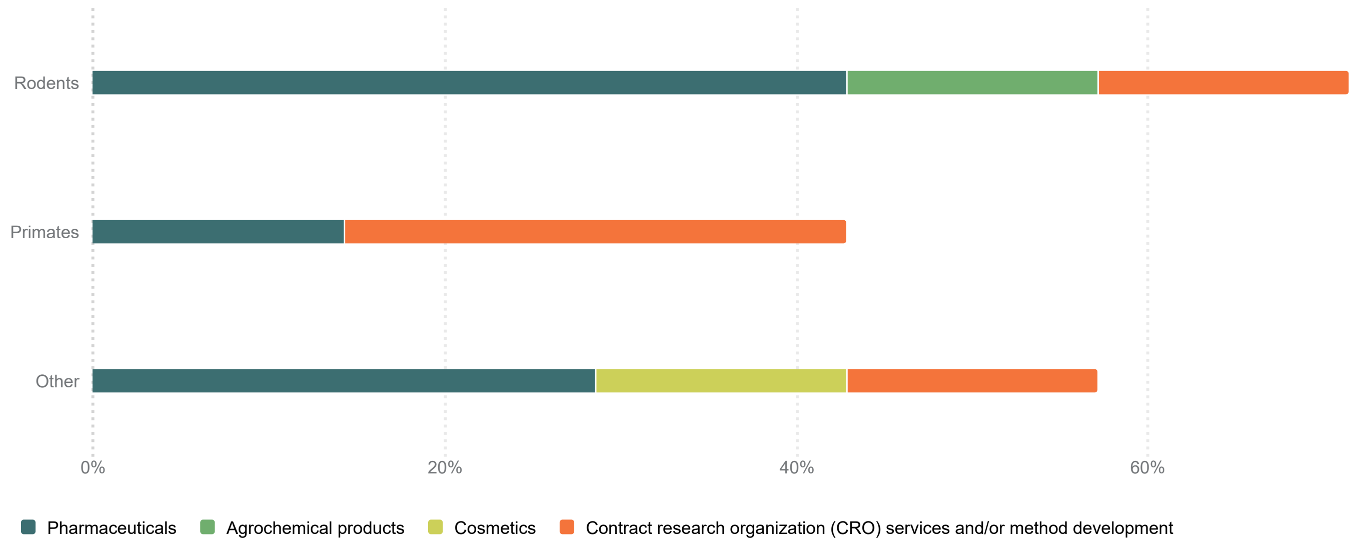

What in vivo models do you use/recommend using to predict human placental toxicity? n=7

Q14 - What in vivo models do you use/recommend using to predict human placental toxicity? - Selected Choice	Percentage	Count
Rodents	71%	5
Primates	43%	3
Other	57%	4

rabbit
dog

9. What is the source of the in vivo models for gonadal toxicity assessment? n=12

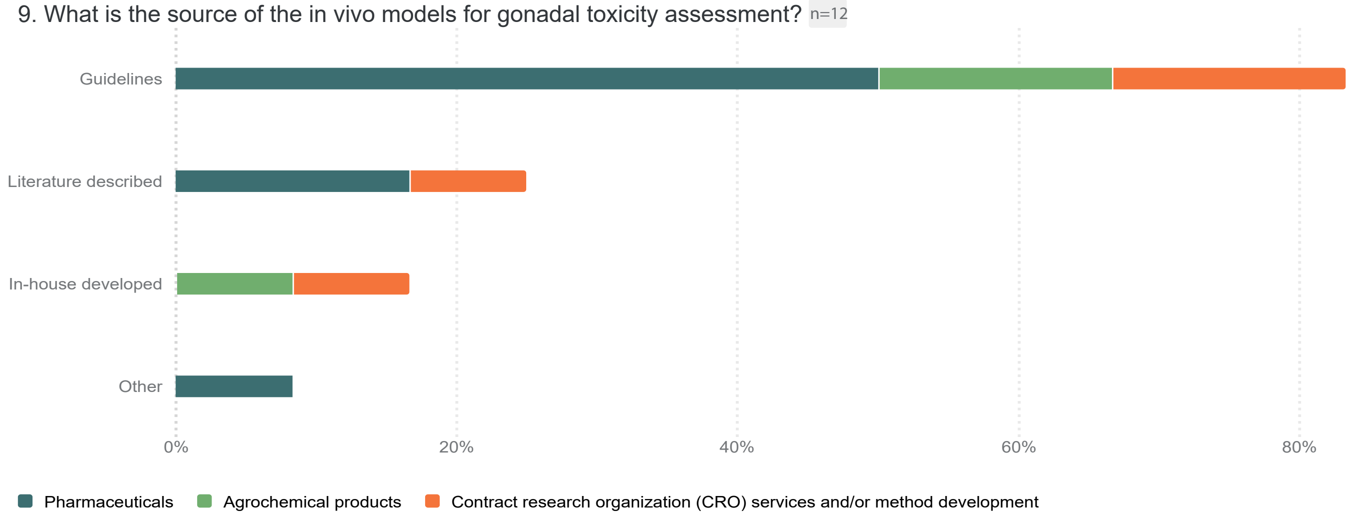

What is the source of the in vivo models for gonadal toxicity assessment? n=12

Q6 - What is the source of the in vivo models for gonadal toxicity assessment? - Selected Choice	Percentage	Count
In-house developed	17%	2
Guidelines	83%	10
Literature described	25%	3
Other	8%	1

10. What is the source of the in vivo models for placental toxicity assessment? n=7

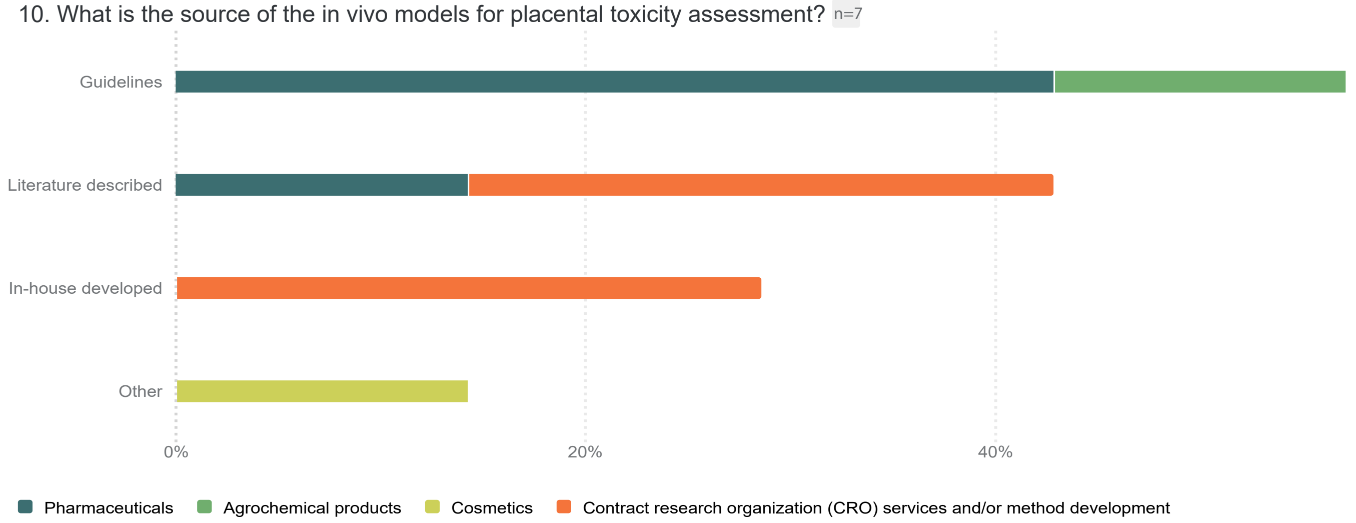

What is the source of the in vivo models for placental toxicity assessment? n=7

Q15 - What is the source of the in vivo models for placental toxicity assessment? - Selected Choice	Percentage	Count
In-house developed	29%	2
Guidelines	57%	4
Literature described	43%	3
Other	14%	1

11. What is your level of confidence in the in vivo models used to conclude impact/safety in human gonads? n=12

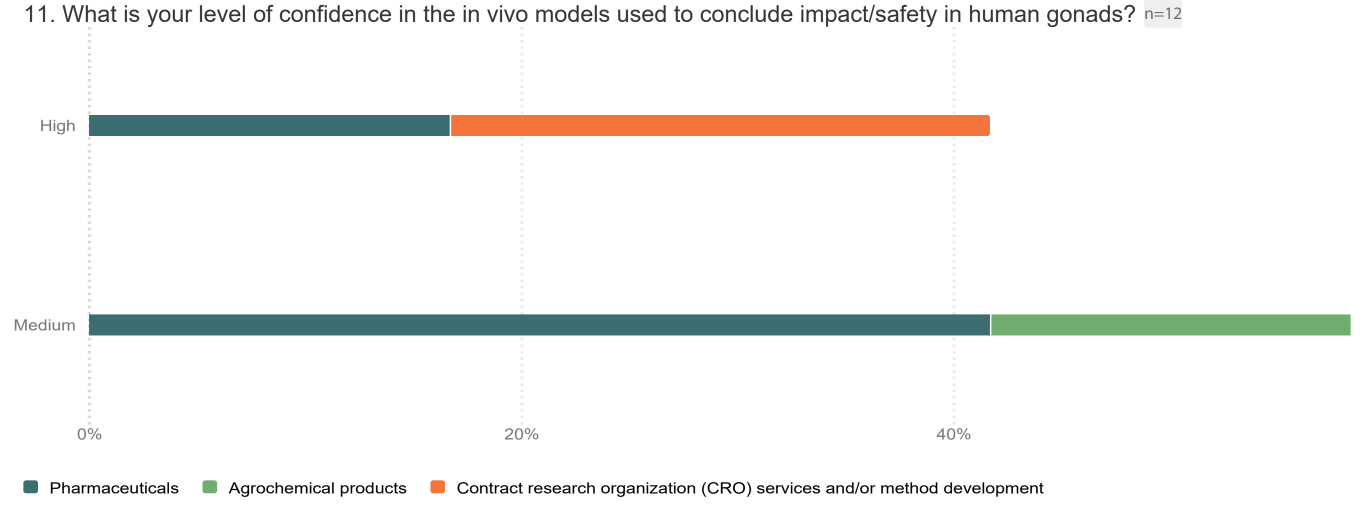

What is your level of confidence in the in vivo models used to conclude impact/safety in human gonads? n=12

Q7 - What is your level of confidence in the in vivo models used to conclude impact/safety in human gonads?	Percentage	Count
High	42%	5
Medium	58%	7

12. What is your level of confidence in the in vivo models used to conclude impact/safety in human placenta? n=7

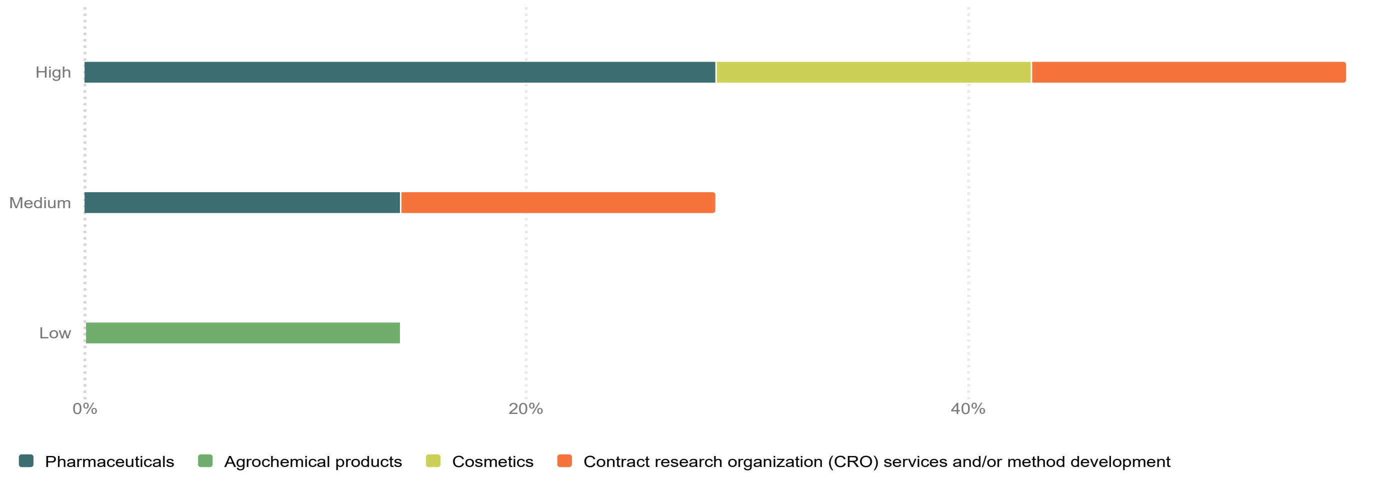

What is your level of confidence in the in vivo models used to conclude impact/safety in human placenta? n=7

Q16 - What is your level of confidence in the in vivo models used to conclude impact/safety in human placenta?	Percentage	Count
High	57%	4
Medium	29%	2
Low	14%	1

13. What makes you choose/recommend these models to evaluate human gonadal toxicity? n=12

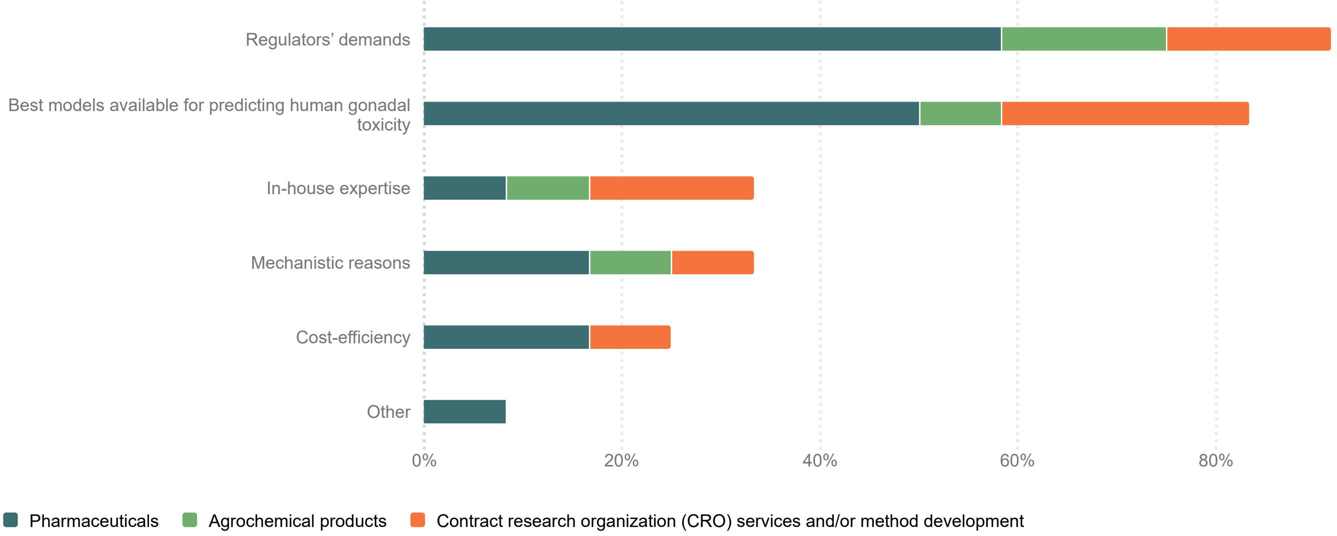

What makes you choose/recommend these models to evaluate human gonadal toxicity? n=12

Q8 - What makes you choose/recommend these models to evaluate human gonadal toxicity? - Selected Choice	Percentage	Count
Regulators' demands	92%	11
Cost-efficiency	25%	3
Best models available for predicting human gonadal toxicity	83%	10
In-house expertise	33%	4
Mechanistic reasons	33%	4
Other	8%	1

14. What makes you choose/recommend these models to evaluate human placental toxicity? n=7

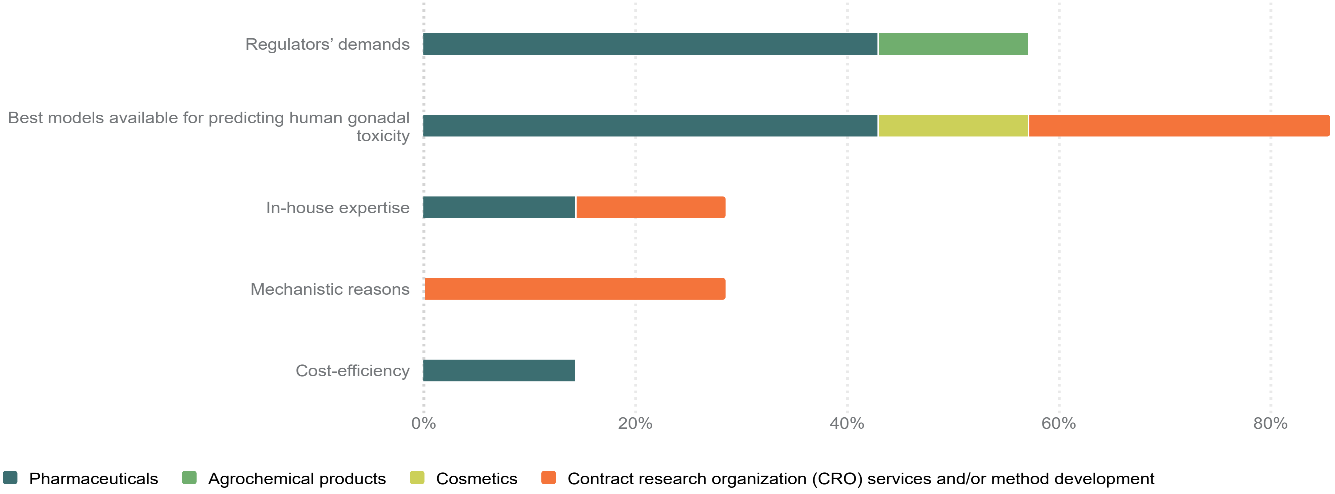

What makes you choose/recommend these models to evaluate human placental toxicity? n=7

Q17 - What makes you choose/recommend these models to evaluate human placental toxicity? - Selected Choice	Percentage	Count
Regulators' demands	57%	4
Cost-efficiency	14%	1
Best models available for predicting human gonadal toxicity	86%	6
In-house expertise	29%	2
Mechanistic reasons	29%	2

15. Do you use/recommend using in vitro NAMs for gonadal toxicity and in which context? n=16

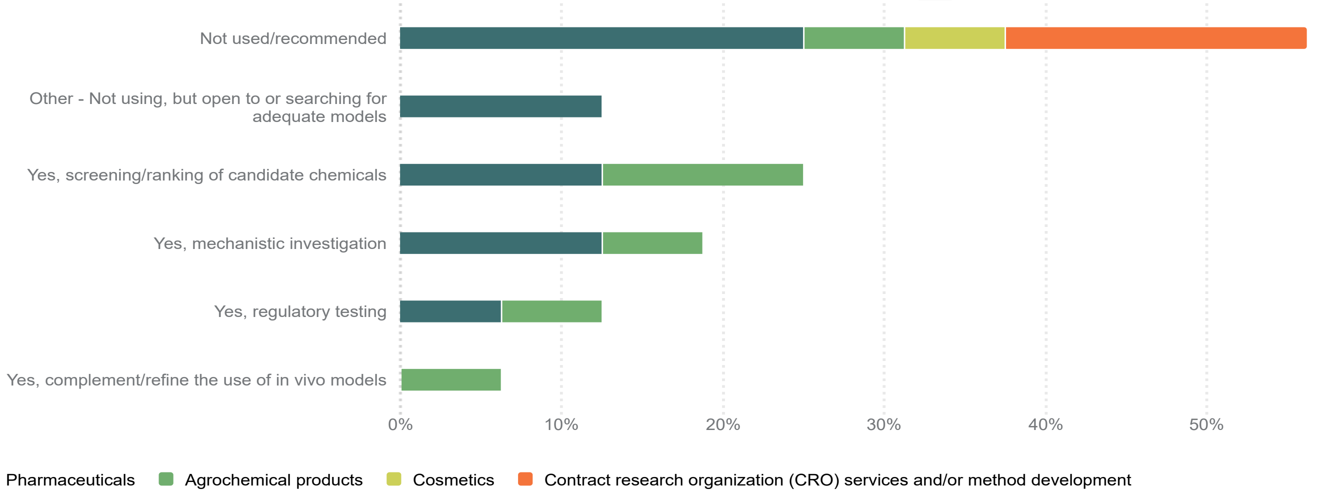

Do you use/recommend using in vitro NAMs for gonadal toxicity and in which context? n=16

Q9 - Do you use/recommend using in vitro NAMs for gonadal toxicity and in which context? - Selected Choice	Percentage	Count
Not used/recommended	56%	9
Yes, regulatory testing	13%	2
Yes, mechanistic investigation	19%	3
Yes, screening/ranking of candidate chemicals	25%	4
Yes, complement/refine the use of in vivo models	6%	1
Other	13%	2

16. Do you use/recommend using in vitro NAMs for placental toxicity and in which context? n=16

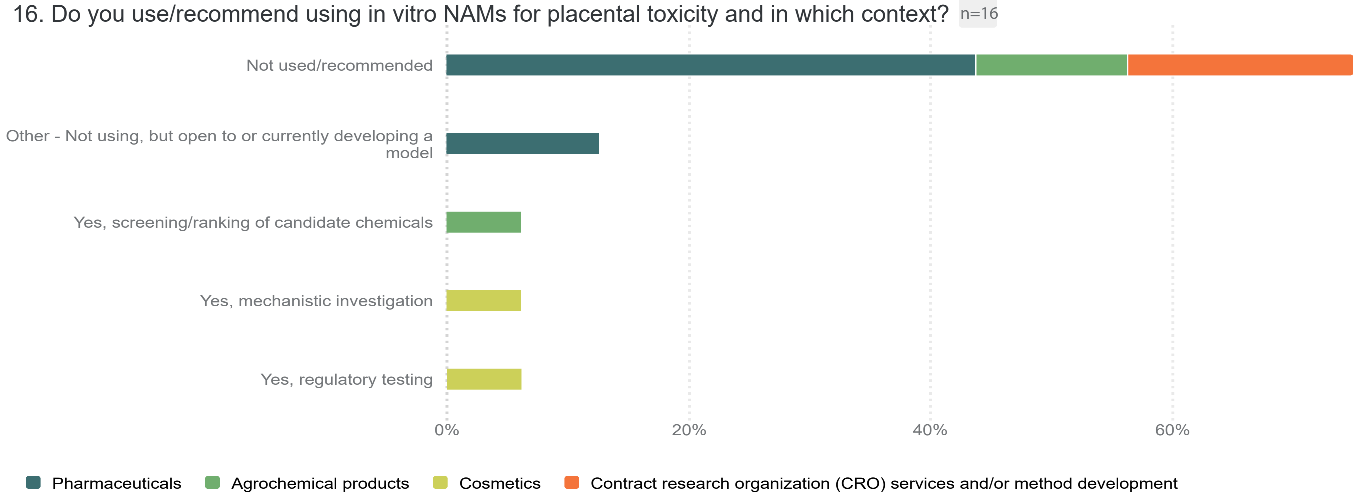

Do you use/recommend using in vitro NAMs for placental toxicity and in which context? n=16

Q18 - Do you use/recommend using in vitro NAMs for placental toxicity and in which context? - Selected Choice	Percentage	Count
Not used/recommended	75%	12
Yes, regulatory testing	6%	1
Yes, mechanistic investigation	13%	1
Yes, screening/ranking of candidate chemicals	19%	1
Other	19%	2

17. What would make you choose/recommend (more) in vitro NAMs to evaluate human gonadal toxicity? n=16

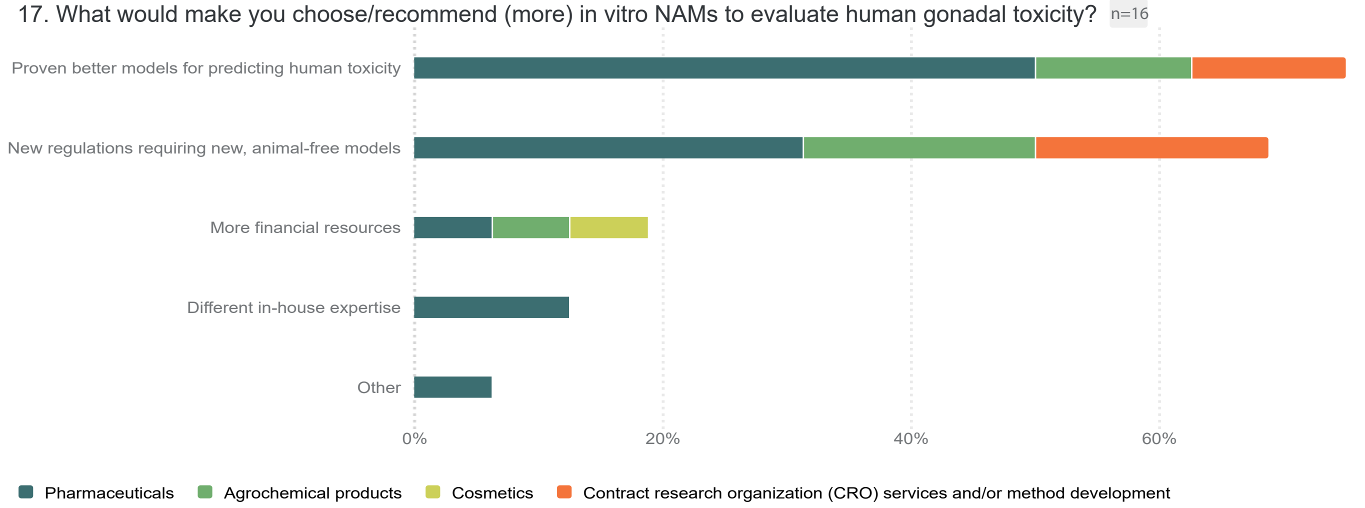

What would make you choose/recommend (more) in vitro NAMs to evaluate human gonadal toxicity? n=16

Q10 - What would make you choose/recommend (more) in vitro NAMs to evaluate human gonadal toxicity? - Selected Choice	Percentage	Count
New regulations requiring new, animal-free models	69%	11
More financial resources	19%	3
Proven better models for predicting human toxicity	75%	12
Different in-house expertise	13%	2
Other	6%	1

18. What would make you choose/recommend (more) in vitro NAMs to evaluate human placental toxicity? n=16

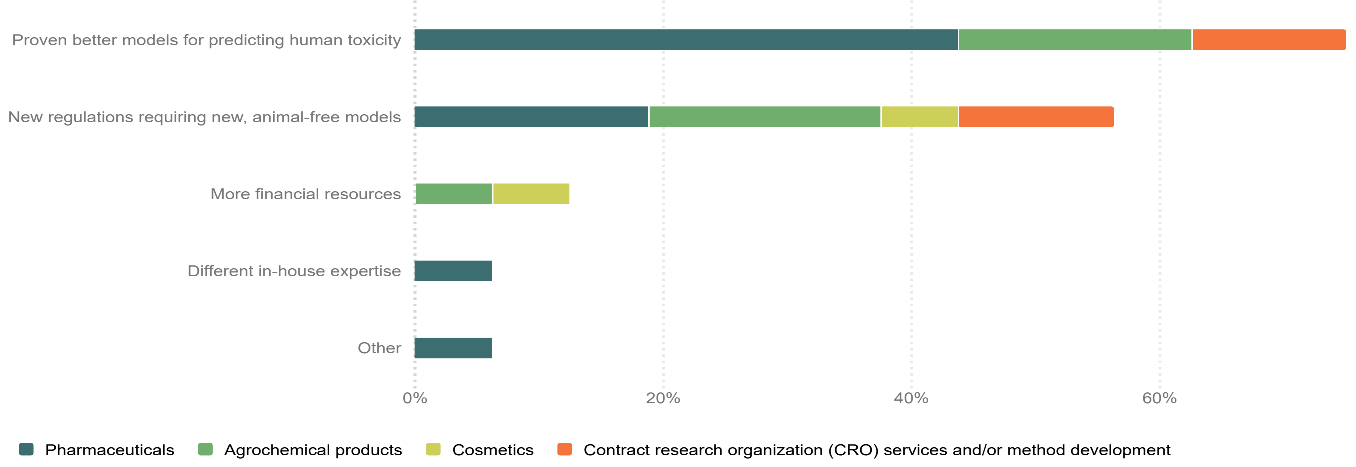

What would make you choose/recommend (more) in vitro NAMs to evaluate human placental toxicity? n=16

Q19 - What would make you choose/recommend (more) in vitro NAMs to evaluate human placental toxicity? - Selected Choice	Percentage	Count
New regulations requiring new, animal-free models	56%	9
More financial resources	13%	2
Proven better models for predicting human toxicity	75%	12
Different in-house expertise	6%	1
Other	6%	1

19. What is your view on using in vitro NAMs in gonadal toxicology for internal decision making? n=16

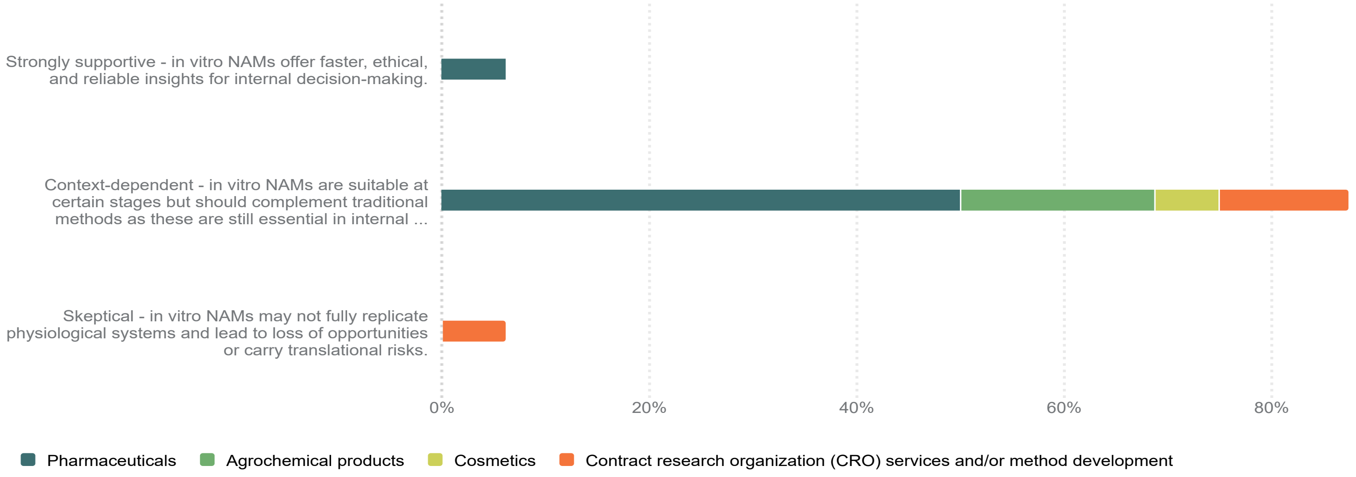

What is your view on using in vitro NAMs in gonadal toxicology for internal decision making? n=16

Q11 - What is your view on using in vitro NAMs in gonadal toxicology for internal decision making? - Selected Choice	Percentage	Count
Strongly supportive - in vitro NAMs offer faster, ethical, and reliable insights for internal decision-making.	6%	1
Context-dependent - in vitro NAMs are suitable at certain stages but should complement traditional methods as these are still essential in internal decision-making.	88%	14
Skeptical - in vitro NAMs may not fully replicate physiological systems and lead to loss of opportunities or carry translational risks.	6%	1

20. What is your view on using in vitro NAMs in placental toxicology for internal decision making? n=16

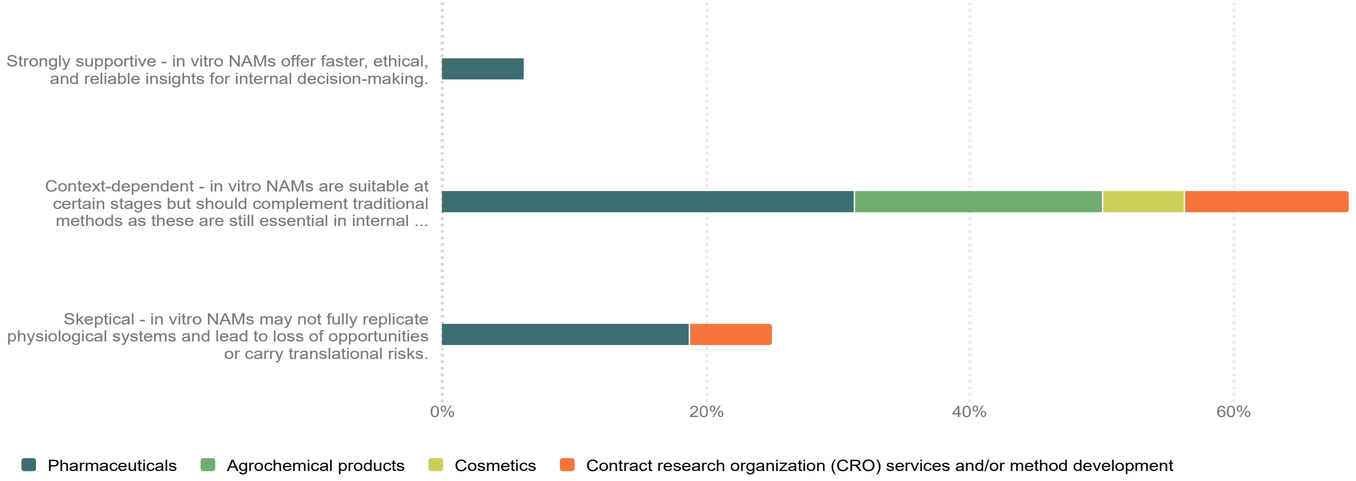

What is your view on using in vitro NAMs in placental toxicology for internal decision making? n=16

Q20 - What is your view on using in vitro NAMs in placental toxicology for internal decision making? - Selected Choice	Percentage	Count
Strongly supportive - in vitro NAMs offer faster, ethical, and reliable insights for internal decision-making.	6%	1
Context-dependent - in vitro NAMs are suitable at certain stages but should complement traditional methods as these are still essential in internal decision-making.	69%	11
Skeptical - in vitro NAMs may not fully replicate physiological systems and lead to loss of opportunities or carry translational risks.	25%	4

21. How soon do you think in vitro NAMs in gonadal toxicology will be used for regulatory purposes? n=16

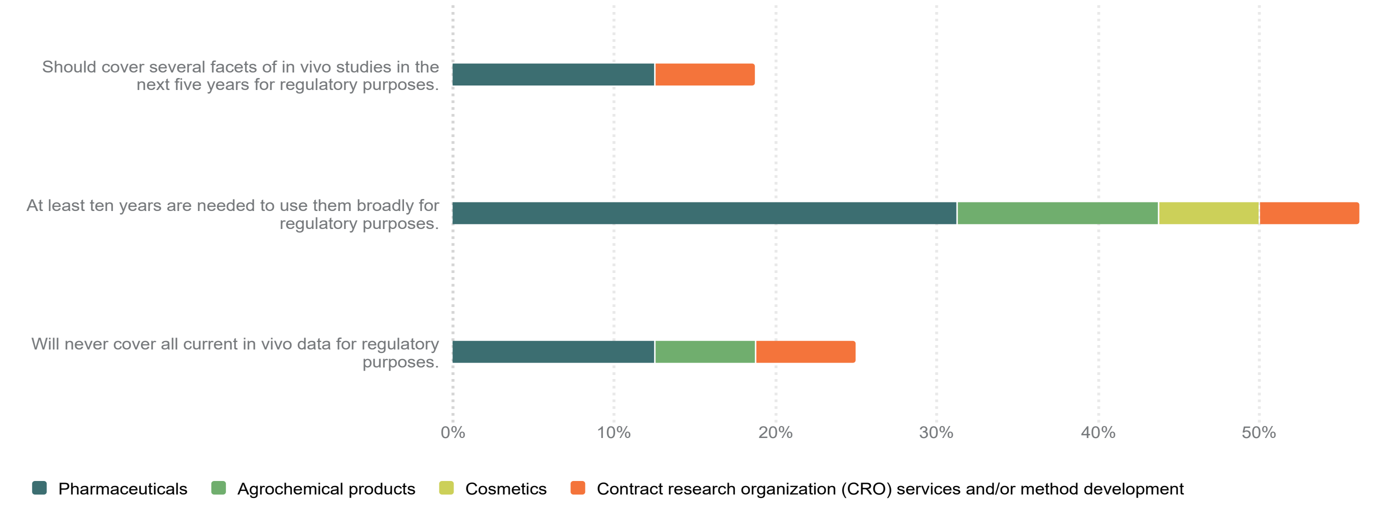

How soon do you think in vitro NAMs in gonadal toxicology will be used for regulatory purposes? n=16

Q12 - How soon do you think in vitro NAMs in gonadal toxicology will be used for regulatory purposes? - Selected Choice	Percentage	Count
Should cover several facets of in vivo studies in the next five years for regulatory purposes.	19%	3
At least ten years are needed to use them broadly for regulatory purposes.	56%	9
Will never cover all current in vivo data for regulatory purposes.	25%	4

22. How soon do you think in vitro NAMs in placental toxicology will be used for regulatory purposes? n=16

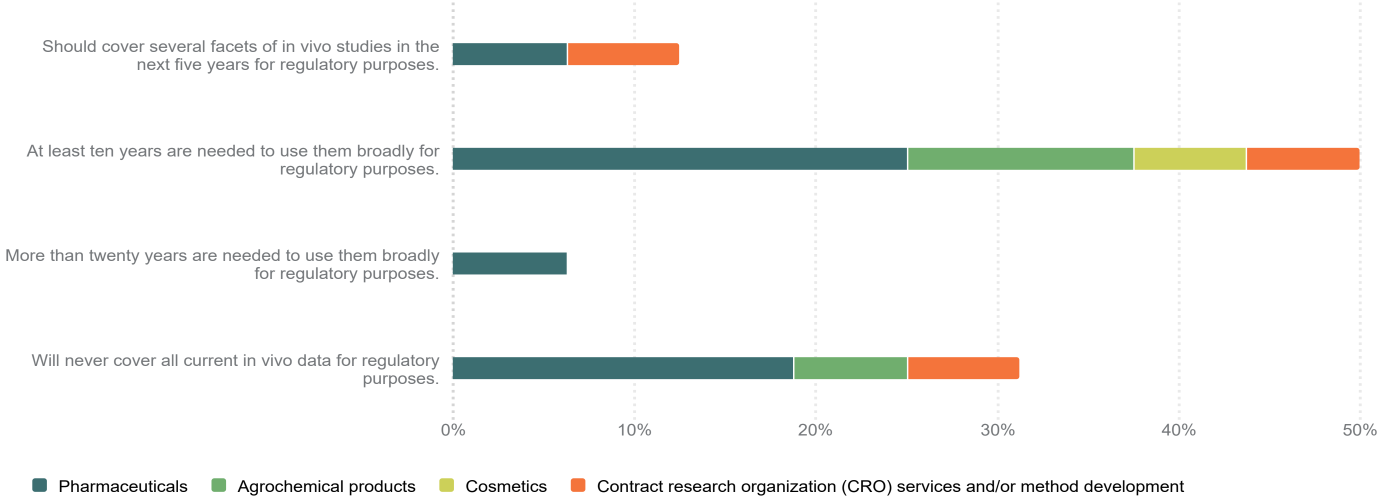

How soon do you think in vitro NAMs in placental toxicology will be used for regulatory purposes? n=16

Q21 - How soon do you think in vitro NAMs in placental toxicology will be used for regulatory purposes? - Selected Choice	Percentage	Count
Should cover several facets of in vivo studies in the next five years for regulatory purposes.	13%	2
At least ten years are needed to use them broadly for regulatory purposes.	50%	8
More than twenty years are needed to use them broadly for regulatory purposes.	6%	1
Will never cover all current in vivo data for regulatory purposes.	31%	5

The following questions were answered only by part of the respondents. Industry sectors represented: pharmaceuticals (n=9), cosmetics (n=1), and CROs (n=2). *Acquaintance* was defined as having used or reviewed data generated from the model. *Confidence* referred to the perceived reliability of the model for safety translation.

23a. Please attribute a personal level of acquaintance to each model type below regarding oogenesis. n=12

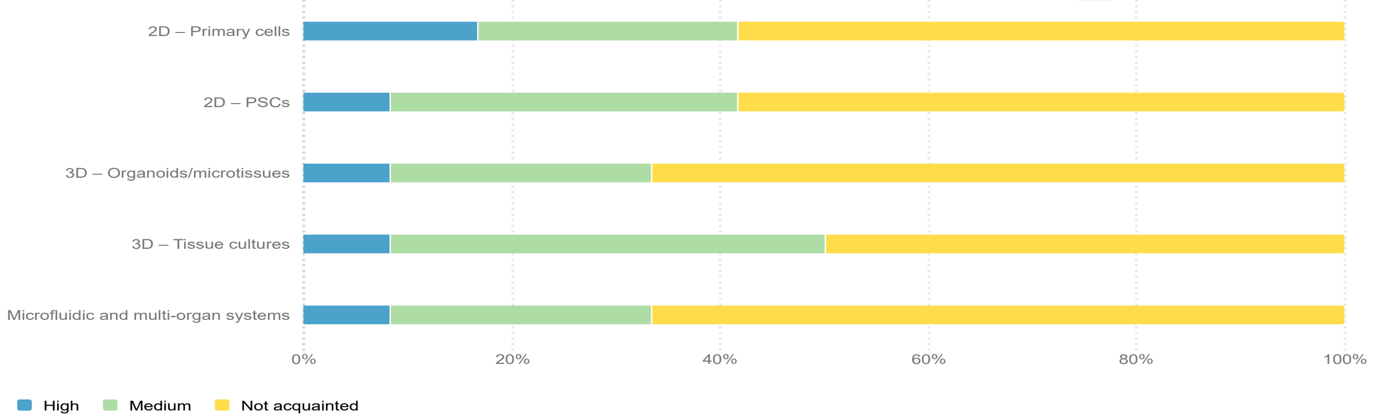

23b. Please attribute a personal level of confidence in safety translation to each model type below regarding oogenesis. n=12

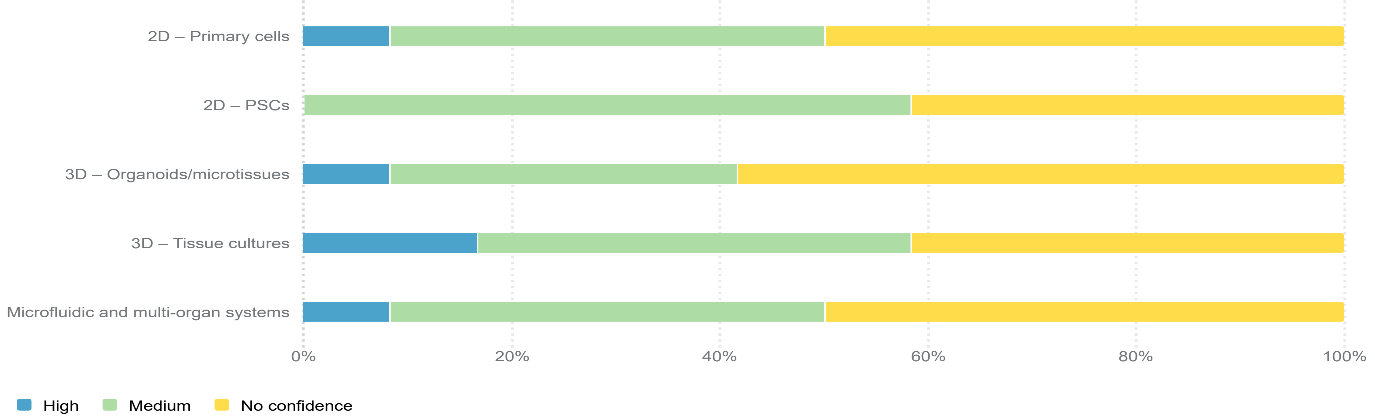

24a. Please attribute a personal level of acquaintance to each model type below regarding spermatogenesis. n=12

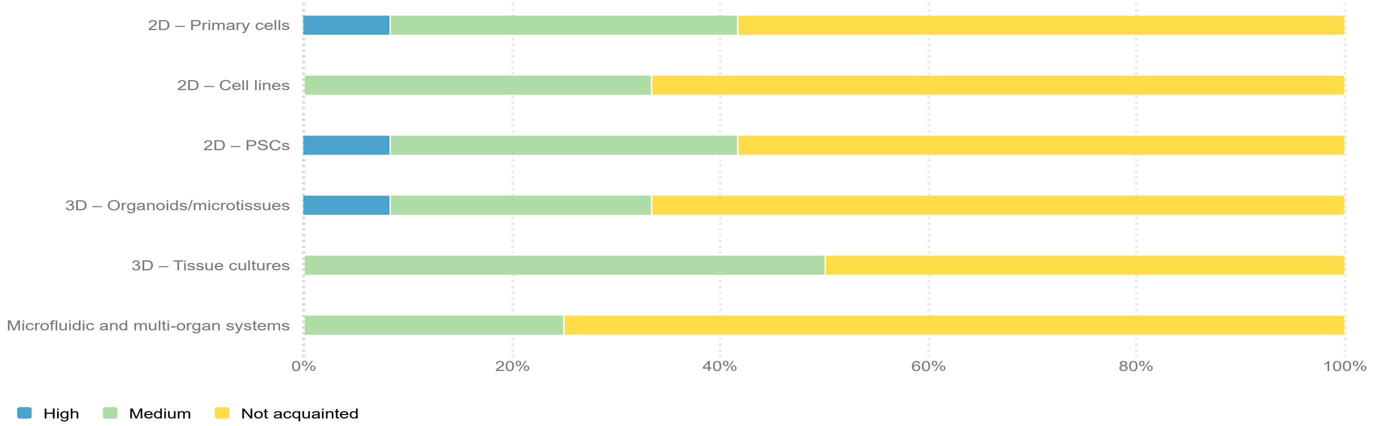

24b. Please attribute a personal level of confidence in safety translation to each model type below regarding spermatogenesis. n=12

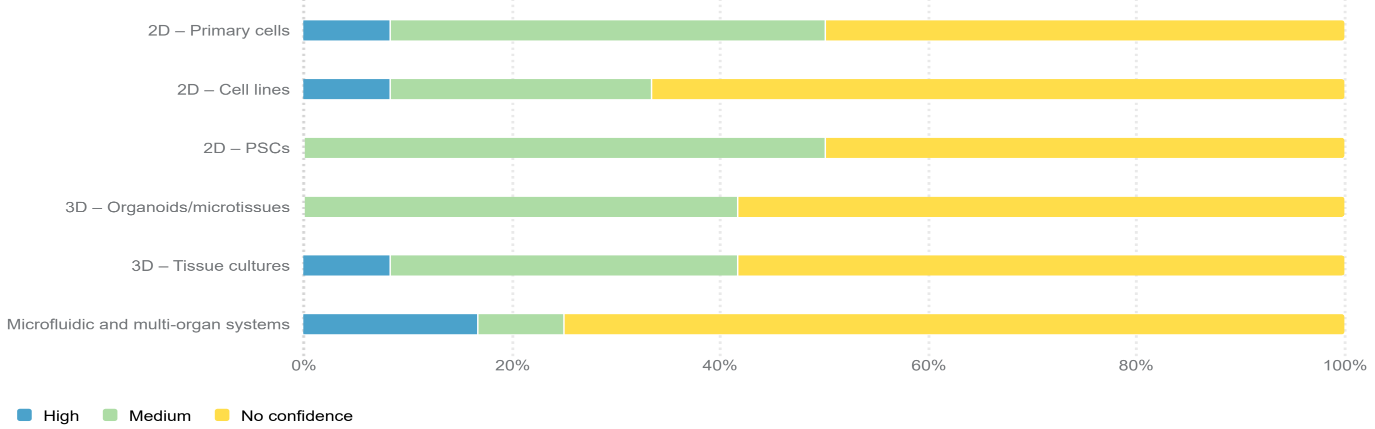

The following questions were answered only by part of the respondents. Industry sectors represented: pharmaceuticals (n=8), cosmetics (n=1), and CROs (n=2).

25a. Please attribute a personal level of acquaintance to each model type below regarding ovarian steroidogenesis. n=11

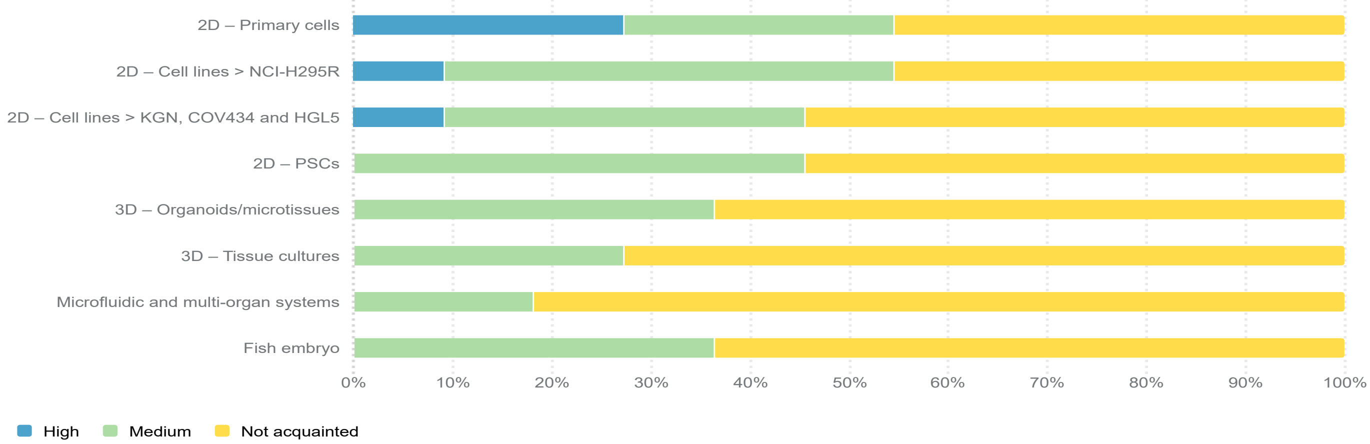

25b. Please attribute a personal level of confidence in safety translation to each model type below regarding ovarian steroidogenesis. n=11

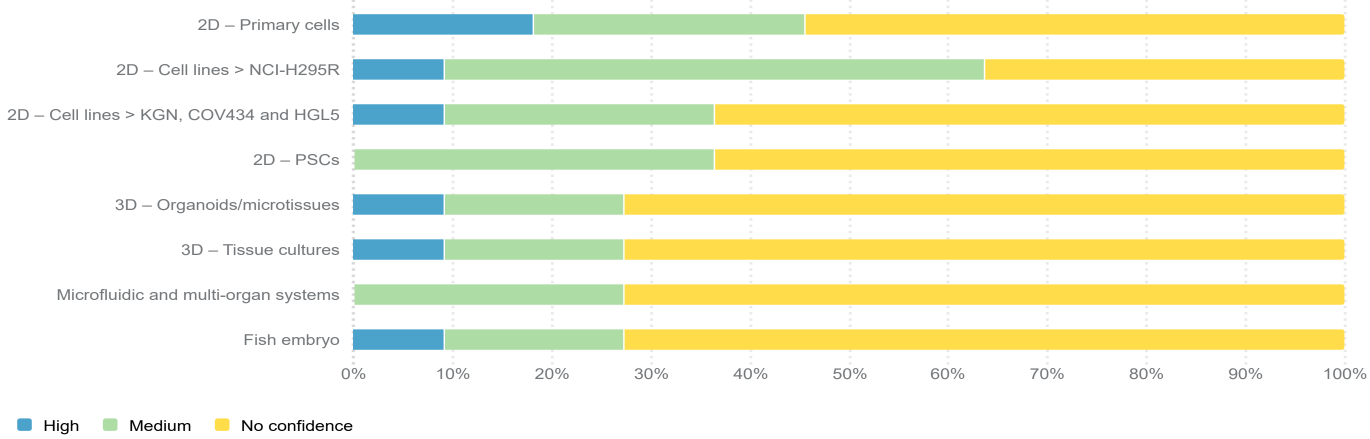

26a. Please attribute a personal level of acquaintance to each model type below regarding testicular steroidogenesis. n=11

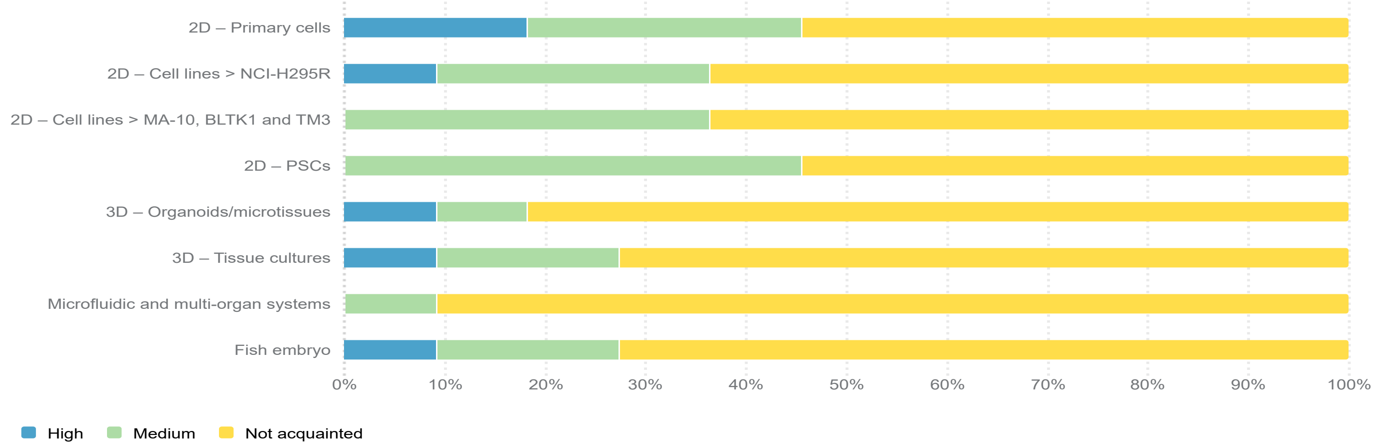

26b. Please attribute a personal level of confidence in safety translation to each model type below regarding testicular steroidogenesis. n=11

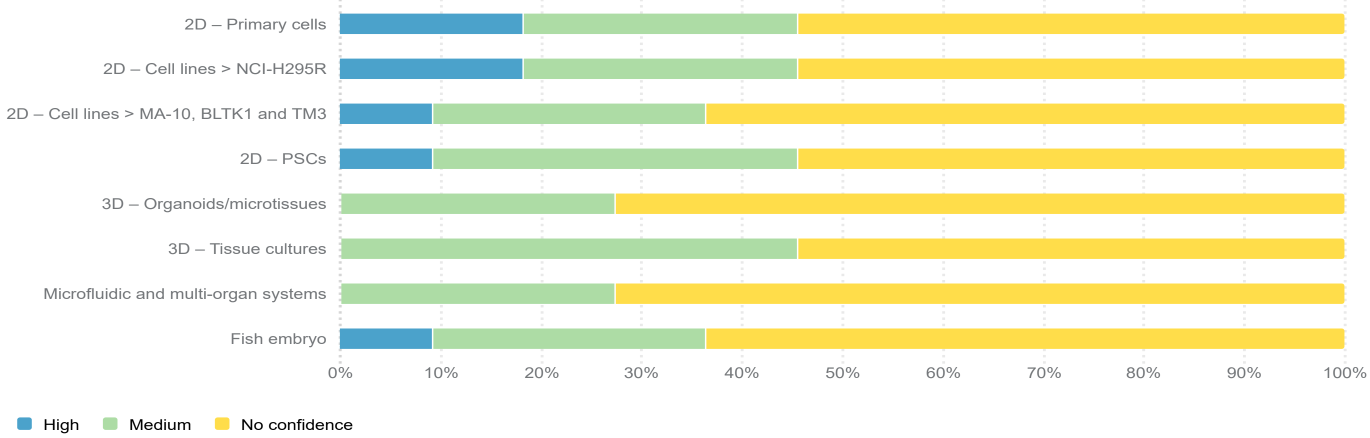

27a. Please attribute a personal level of acquaintance to each model type below regarding placental function. n=11

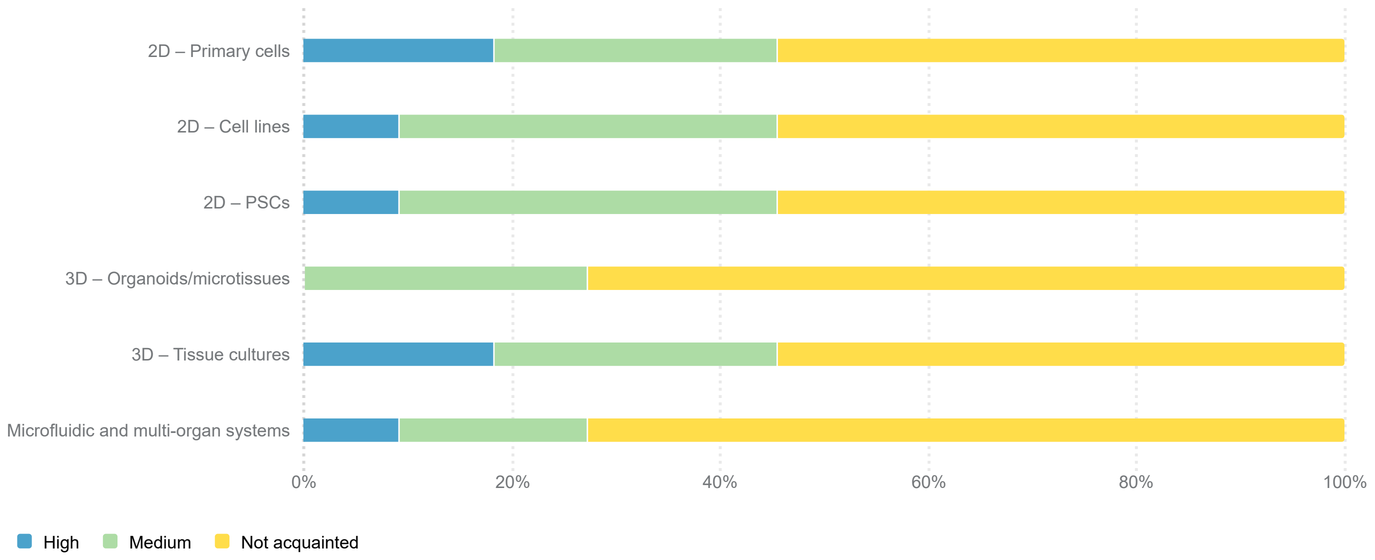

27b. Please attribute a personal level of confidence in safety translation to each model type below regarding placental function. n=11

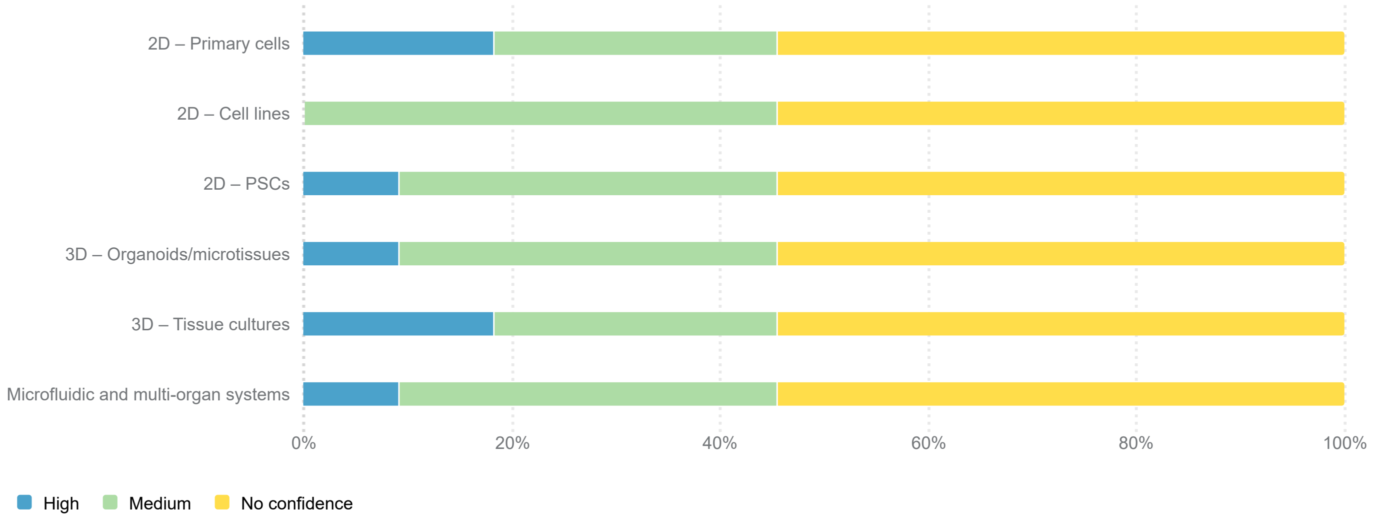

Supplement: Supplementary file 1 — A supplementary file is available with the survey details, questions and responses. [file mmc1.pdf]
